# guidedNOMe-seq quantifies chromatin states at single allele resolution for hundreds of custom regions in parallel

**DOI:** 10.1186/s12864-024-10625-3

**Published:** 2024-07-29

**Authors:** Michaela Schwaiger, Fabio Mohn, Marc Bühler, Lucas J. T. Kaaij

**Affiliations:** 1 Friedrich Miescher Institute for Biomedical Research, Basel, 4056 Switzerland; 2 Swiss Institute of Bioinformatics, Basel, 4056 Switzerland; 3 University of Basel, Basel, 4003 Switzerland; 4 Center for Molecular Medicine, University Medical Center Utrecht, Utrecht, 3584 CG The Netherlands

**Keywords:** NOMe-Seq, Cas9 enrichment, Chromatin, CTCF, ChAHP

## Abstract

Since the introduction of next generation sequencing technologies, the field of epigenomics has evolved rapidly. However, most commonly used assays are enrichment-based methods and thus only semi-quantitative. Nucleosome occupancy and methylome sequencing (NOMe-seq) allows for quantitative inference of chromatin states with single locus resolution, but this requires high sequencing depth and is therefore prohibitively expensive to routinely apply to organisms with large genomes. To overcome this limitation, we introduce guidedNOMe-seq, where we combine NOMe profiling with large scale sgRNA synthesis and Cas9-mediated region-of-interest (ROI) liberation. To facilitate quantitative comparisons between multiple samples, we additionally develop an R package to standardize differential analysis of any type of NOMe-seq data. We extensively benchmark guidedNOMe-seq in a proof-of-concept study, dissecting the interplay of ChAHP and CTCF on chromatin. In summary we present a cost-effective, scalable, and customizable target enrichment extension to the existing NOMe-seq protocol allowing genome-scale quantification of nucleosome occupancy and transcription factor binding at single allele resolution.

**Supplementary Information:**

The online version contains supplementary material available at 10.1186/s12864-024-10625-3.

## Background

To determine the gene regulatory elements that are active in a cell at any given moment is a major challenge [1–4]. At the same time a deeper understanding of gene regulatory elements is of pivotal importance, since understanding their activity during development will ultimately allow their specific manipulation, which could potentially open novel therapeutic avenues [5–9]. Epigenomic maps stratify the genome-wide chromatin state with ever increasing resolution. For instance, ChIP-seq can be used to determine which part of the genome shows enhancer-like characteristics or is bound by transcription factors (TFs) of interest. ATAC-seq and DNaseI hypersensitivity assays map regions of the genome that are “open” and therefore accessible to DNA binding factors and the transcriptional machinery, whereas MNase-seq is used to determine nucleosome positioning [10]. These techniques, initially used in bulk on hundreds-to-millions of cells, have all been adapted to measure chromatin states in single cells [9]. Unfortunately, these single cell measurements result in sparse datasets making reliable quantification of single locus-TF combinations very challenging and due to the requirement of high sequencing depth also very expensive [11]. These techniques are enrichment based and report on the relative frequencies a certain locus is, for instance, TF bound, but lacks information about the number of times a locus is not bound. This makes the data semi-quantitative. The semi-quantitative nature of such data sets results in the necessity to perform different normalization methods for differential binding analyses depending on the biological scenario. Importantly, the correct normalization method is not always obvious, but does greatly impact the outcome [12].

**Fig. 1:**
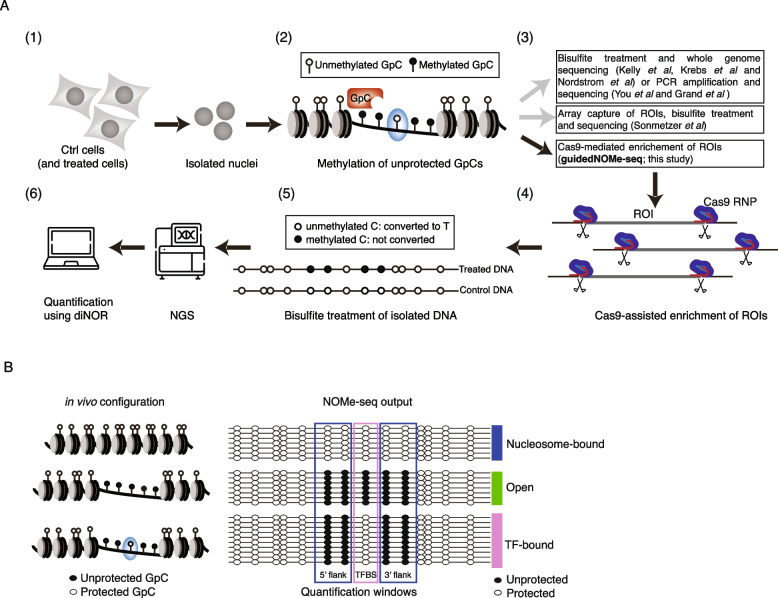
NOMe-seq approaches. **A** Schematic overview of the in situ M.CviPI treatment that lays at the basis of the NOMe-seq protocol. From left to right. (1) Cells are harvested, and nuclei are isolated using a cell lysis buffer. (2) Intact nuclei are incubated with M.CviPI. M.CviPI specifically methylates cytosines present in the GpC context that are accessible (e.g. not TF or nucleosome bound). (3) After M.CviPI treatment the genomic DNA is extracted and bisulfite treated. Different experimental strategies that are currently used to process the genomic DNA before sequencing. (4) Schematic representation of guidedNOMe-seq using Cas9 in combination with sgRNA pools to liberate hundreds of ROI in parallel from intact genomic DNA. (5) Sequencing of bisulfite treated DNA is used to determine which cytosines are methylated and which are not. At this step endogenous methylation and M.CviPI mediated methylation can been distinguished as this occurs in different sequence contexts. (6) Next generation sequencing (NGS) of the guidedNOMe libraries followed by in depth analysis using differential NOMe R package. **B** (left) Schematic representation how different chromatin states (TF bound, Open and Nucleosome bound) of a single locus result in different M.CviPI methylation patterns. (right) Schematic representation of a single ROI with 24 single DNA molecule coverage. Using the observed protected and unprotected states of GpCs present in the three chromatin state quantification windows, the chromatin state of individual DNA molecules can be classified

Nucleosome occupancy and methylome sequencing (NOMe-seq) is an elegant method that yields quantitative genome-wide single DNA molecule information regarding nucleosome positioning, chromatin accessibility, TF binding and endogenous DNA methylation [13, 14]. In the NOMe-seq protocol, nuclei are isolated from cells and incubated with a GpC methyltransferase (M.CviPI) (Fig. 1A). M.CviPI methylates cytosines only when present in the GpC context, which makes it possible to discriminate these sites from endogenous cytosine methylation that only occurs in the CpG context in vertebrate cells. Upon addition of M.CviPI to the isolated nuclei, cytosines in the GpC context that are accessible to the methyltransferase will be methylated, whereas DNA bound by nucleosomes or TFs will not be accessible and therefore remain unmethylated. Following in situ M.CviPI methylation, genomic DNA is isolated and the methylation status of the cytosines is determined using bisulfite treatment and subsequent sequencing of either individual regions of interest (ROI) or genome-wide (Fig. 1A).

Reliable quantification of chromatin states with single ROI resolution requires high coverage, i.e., > 30-fold, and ideally long reads in order to quantify regions encompassing multiple nucleosomes and TF-binding sites. Due to the large size of vertebrate genomes, achieving such coverage genome-wide is very costly. Approaches reducing complexity and/or enriching for parts of the genome have been developed to achieve the required coverage at reasonable costs. For example, single ROI can be amplified using conventional PCR followed by Sanger or deep sequencing [15, 16]. Such PCR-based enrichments result in high ROI coverage with informative reads, but at the expense of throughput. Moreover, PCR amplification and primer design for bisulfite treated DNA is challenging and can result in unwanted biases [17]. Recently, Sönmezer et al. circumvented the coverage issue by an enrichment strategy using commercially available capture arrays. This resulted in high coverage over a small (~ 2%) part of the genome [14]. While this strategy works well for TSSs and putative cis-regulatory elements and enhancers present on the capture-array, a major drawback of commercially available arrays is the lack of ROI flexibility, the restriction to the human and mouse genome, and inherent difficulties of assessing repetitive regions.

A second major limitation of this approach is the need for chromatin shearing, which results in heterogeneous fragments that cover the ROI only partially. Throughout this manuscript we infer the chromatin state of individual DNA molecules, i.e. nucleosome bound, TF bound or open, using the combined methylation information in three adjacent windows as introduced by Sönmezer et al. [14]. Hence, for this analysis to be meaningful, each single sequencing read needs to span all three windows which encompass at least hundred base-pairs (Fig. 1B). If DNA is randomly sheared, such as for whole-genome or array-capture NOMe-seq, this results mostly in DNA fragments that do not fully cover the ROI, which won’t be useful for analysis (see below). We note that this classification strategy precludes the identification of loci that are actively being remodeled and using our cut-offs these molecules are most likely classified as nucleosome bound.

Nevertheless, NOMe-seq has been demonstrated to be a very powerful technique. For instance, it was used to investigate the cooperativity of TF binding to chromatin and quantification of endogenous DNA methylation in combination with TF binding at single molecule resolution [14, 18]. Combined with long-read sequencing, NOMe-seq has been used to gain high-resolution allele-specific chromatin state information for a hand full of loci [19]. NOMe-seq has also been used to quantify chromatin states in single cells [20, 21]. Yet, despite the high quality of data, NOMe-seq has so far not been widely adopted by the chromatin community, which might be in part due to the above discussed technological limitations and the scarcity of computational tools to analyze NOMe-seq data.

To overcome some of these limitations and inspired by recent reports utilizing Cas9 as a specific programmable nuclease to enrich or deplete ROI in sequencing libraries, we developed guidedNOMe-seq [22–24]. guidedNOMe-seq is a cost-effective extension of classical NOMe-seq that allows for enrichment of hundreds to thousands of ROI from the genome of any organism. This is achieved by making use of a custom ROI-specific sgRNA pool loaded in vitro on recombinant Cas9 for efficient ROI liberation from genomic DNA (Fig. 1A). Further, we have developed a differential NOMe (dinoR) R package to facilitate data visualization and the statistical comparison of different NOMe-seq samples.

Taken together, guidedNOMe-seq is a quantitative, highly customizable and cost-effective assay to profile chromatin occupancy of nucleosomes and TFs at single molecule resolution that will help to decipher the mechanistic and time-resolved steps of chromatin-templated events.

## Results

### Design of a guidedNOMe-seq experiment

**Fig. 2:**
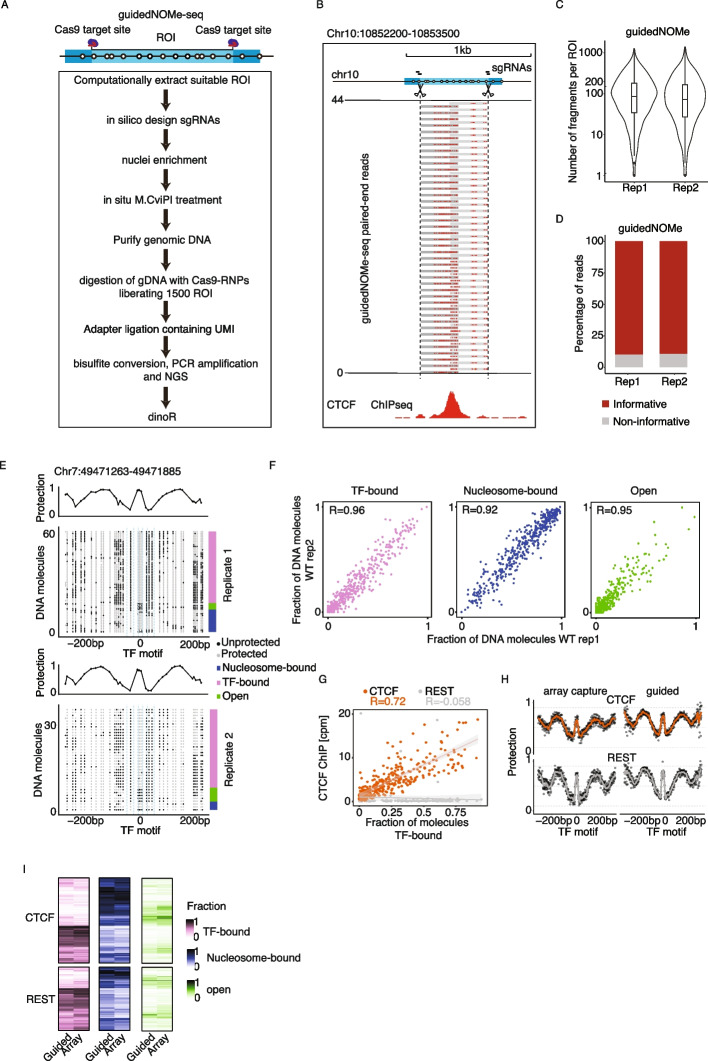
Benchmarking guidedNOMe-seq. **A** Overview of the guidedNOMe-seq protocol. (1) Computationally determine which ROI contain GpC in the required windows. (2) Design pairs of sgRNAs targeting your ROI. (3) Perform in situ M.CviPI treatment, extract genomic DNA and perform sgRNA-Cas9 mediated custom digest. (4) Ligate methylated adapters to the digested DNA, perform bisulfite conversion, PCR amplify and check size distribution on bioanalyzer. **B** Genome browser view of a ROI showing from top to bottom: (top) the position of the 4 sgRNAs designed up and downstream of the ROI, (middle) guidedNOMe-seq read coverage at the target locus and (bottom) CTCF ChIPseq. **C** Violin plot showing the distribution of fragment coverage over individual ROI in the two guidedNOMe-seq replicates. **D** Density plot showing the percentage of informative (spanning the three chromatin state quantification windows) reads when performing guidedNOMe-seq. **E** Single ROI guidedNOMe-seq example of a CTCF binding site and its surrounding (~ 250 bp ±). Top plot shows the average protection value of the GpCs at the indicated location. Bottom plot shows protected and unprotected state of the GpCs in individual DNA molecules. DNA molecules are sorted based on the chromatin state they are in, as indicated in the barplot on the right. **F** Scatterplots showing the reproducibility between replicates of the chromatin state classification. Every ROI is depicted as a single dot. Only ROI with at least 30 informative fragments are shown. **G** Scatter plot showing the guidedNOMe-seq inferred fraction of TF bound DNA molecules per ROI split by replicate (x-axis) and CTCF ChIPseq signal in ChIP replicate 1 (y-axis). **H** Comparing the average protection profiles as observed with the Array capture and guidedNOMe-seq data. The y-axis shows the average protection a GpC position has against exogenous methylation by M.CviPI (1-mGpC). LOESS line is added through the individual data points and ROI are split on TF. Only ROI that are shared between the two enrichment techniques are used. **I** Comparing the TF chromatin state classification as observed in the array capture and guidedNOMe-seq data. ROI are split on TF. Only ROI that are shared between the two enrichment techniques are used

For guidedNOMe-seq we start out with a group of ROI, for example binding sites of a specific transcription factor (TF) that are subject to investigation in the respective study and, importantly, unrelated ROI, e.g. binding sites of an unrelated TF under the respective experimental conditions, that can be used as internal technical controls. The guidedNOMe-seq procedure entails the following steps (Fig. 2A): 1) Computational identification of ROI including filtering for the presence of GpCs in the three chromatin quantification windows. 2) Selection of suitable Cas9 cut sites up and downstream of the ROI chromatin quantification windows. It is important to select the Cas9 cut sites in such a way that all three chromatin quantification windows are sequenced from a single DNA molecule. The precise parameters depend on the sequencing platform and modality used. In this study, we made use of Illumina paired-end 300 bp sequencing and thus were able to choose the ROI spanning 600 bp surrounding the TF motif center, with Cas9 targeting a window of 80 bp at the 5’ and 3’ end of the ROI. This resulted in library insert sizes ranging from 440 to 600 bp, covering 3 nucleosomes on average. 3) Execution of the standard NOMe-seq protocol [16] until the isolation of genomic DNA. 4) Performing custom Cas9 digest using the in silico designed sgRNA pool specific to the ROI. 5) Ligation of methylated adapters (compatible with the respective sequencing technology used) containing unique molecular identifiers (UMIs) to the NOMe-treated Cas9-digested genomic DNA fragments. 6) Bisulfite conversion of the library followed by PCR amplification and sequencing on the platform of choice. 7) Alignment to bisulfite-aware reference genome and quantification of chromatin states.

Of note, this protocol results in a library containing a custom set of ROI with 5’ and 3’ ends defined by the Cas9 cut sites and thus allows simple estimation of ROI enrichment and hence quality of the acquired data.

### Technical aspects of guidedNOMe-seq

To benchmark guidedNOMe-seq, we generated two biological replicate guidedNOMe-seq libraries from wild-type mouse embryonic stem cells (mESC) targeting 1226 ROI with binding sites for the well-studied transcription factor CTCF and 274 ROI entailing binding sites for the transcription factor REST.

Visual inspection and computational analysis of the guidedNOMe-seq data showed that the ROI enrichment works efficiently and that the sequencing reads start at the expected Cas9 cut sites (Fig. 2B). Notably, only 1.3% of ROI (20 out of 1500) showed no read coverage in both replicates, while for the vast majority fragment coverage over ROI is relatively homogenous and comparable between replicates (Figs. 2C and S1A). Moreover, the high correlation between replicates shows that the Cas9-sgRNA RNPs reproducibly enrich hundreds of ROI from a complex genome and that either ROI intrinsic or experimental parameters (e.g. Cas9 cutting efficiency) dictate fragment coverage (see below). Of the 4 and 5.5 million paired-end reads (which equals genomic coverage of ~ 1) sequenced in the two replicates, we found 197 k and 186 k reads spanning the selected ROI which corresponds to a 140- and 100-fold enrichment, respectively (see Methods). Importantly, due to the defined 5’ and 3’ cut sites in guidedNOMe-seq, 90% of reads that map to the ROI span all three chromatin quantification windows and are therefore usable to quantify chromatin states (Fig. 2B and D). The unique molecular identifiers (UMIs) present in the ligated adapters allow for the identification and removal of PCR duplicates. Plotting deduplicated fragment counts versus duplicated fragment counts shows that there are indeed PCR duplicates, indicating that the libraries are sequenced to saturation and demonstrating that the addition of UMIs is essential for such quantitative experimental approaches (Figure S1B). This analysis also confirms that UMIs are distributed equally over all ROI and scale proportionally to the coverage of the ROI, suggesting that there are no major locus-specific ligation or PCR biases for the over thousand different genomic regions.

We next generated single ROI plots to assess reproducibility between biological replicates. Reassuringly, both replicates produce very similar nucleosome and TF footprints (Fig. 2E). Further, when globally comparing the chromatin states called based on the footprint patterns (TF, nucleosome occupied, or open, see Fig. 1B) in all suitable ROI with a coverage of at least 30 reads, we found that guidedNOMe-seq results in highly reproducible chromatin state classifications (Fig. 2F). To further validate the chromatin state classifications, we compared the TF chromatin state quantification of all ROI with CTCF ChIP-seq signal. This has previously been reported to correlate, indicating that CTCF ChIP-seq enrichment is a reasonable proxy for TF occupancy at individual binding sites [14]. As expected, the control group consisting of REST ROI shows no correlation with CTCF ChIP-seq enrichment, whereas the CTCF ROI show a linear correlation between the TF state quantified by guidedNOMe-seq and CTCF ChIP-seq (*r* = 0.72) (Fig. 2G).

From these analyses we conclude that guidedNOMe-seq is a robust, specific, and versatile extension of existing NOMe-seq protocols that allows unique customization at a medium throughput.

### Comparison with other ROI enrichment methods

We next set out to compare guidedNOMe-seq to other locus specific or genome-scale approaches. NOMe followed by locus-specific PCR-based analysis achieves high specificity and coverage and is therefore considered the gold standard (Figure S1D). It also results in defined 5’ and 3’ ends and thus a high percentage of informative reads, as observed for guidedNOMe-seq (Fig. 2D). However, it suffers from two major drawbacks: First, it is not easily scalable to hundreds or thousands of ROI. Secondly, it is prone to PCR biases and artifacts as primer design and PCR amplification using bisulfite treated DNA is inherently difficult [17, 25].

Another strategy to reduce complexity and thus sequencing costs is to combine NOMe-seq with commercial array capture-based enrichment as introduced by Sönmezer et al. [14]. This strategy is comparable to guidedNOMe-seq but offers less flexibility and customizability. For example, the commercial array capture probes used in the Sönmezer et al. study target ~ 2% of the mouse genome with a focus on regulatory regions such as transcription start sites (TSSs) and cis-regulatory elements (CREs) but were not specifically designed to enrich TSS distal ROI or TF binding sites. The ROI coverage between replicates is comparable with guidedNOMe-seq (Figure S1C), but the percentage of mapped reads overlapping with ROI is considerably higher when using array capture (90%) as compared to Cas9-mediated enrichment (23%; Figure S1D). However, many of those on-target sequencing reads from the array capture approach cannot be used for inferring chromatin states of individual loci because they do not span the three quantification windows. This is due to a simple technical reason: The array capture protocol relies on shearing of genomic DNA prior to hybridization, which is commonly performed by sonication. This leads to randomly sheared genomic fragments without defined 5’ and 3’ ends. Because of this, ~ 80% of the on-target reads do not span all three chromatin quantification windows. Consequently, these reads cannot be used for chromatin state quantification (Figure S1E). In addition, the inflexible commercial array design results in the enrichment of regions that are not necessarily of value to address a particular research question.

Taken together, whereas the guidedNOMe-seq protocol generates more off-target reads, array capture NOMe-seq yields more non-usable reads on target, which ultimately results in comparable percentages of reads that can be used for chromatin state quantification between techniques. To put this in numbers for our specific set of ROI and when we only look at the 465 ROI shared between the guidedNOMe-seq generated here and the published array capture NOMe-seq dataset [14]. If we wanted to achieve that ~ 60 percent of the ROI shared between guided and array capture libraries have at least 30 × informative read coverage, we would need to sequence ~ thirty times more reads (125–150 million vs 4–5.5 million reads) in an array capture approach in comparison to guidedNOMe-seq due to the high number of non-informative ROI when studying a specific TF like CTCF.

The 465 ROI that are shared between our guidedNOMe-seq data and the array capture NOMe-seq have a similar amount of reads at the different sequencing depths used (~ 50 k for guided, ~ 50 k-150 k for array). When comparing average protection profiles over CTCF and REST binding sites, the two methods reveal very similar average protection patterns over all ROI (Fig. 2H). Compared to methods like ChIP-, MNase- or ATAC-seq, an advantage of NOMe-seq is the ability to quantify chromatin states with single DNA molecule resolution. To determine if guidedNOMe-seq also compares well with the array capture NOMe-seq at this level, we quantified chromatin states with single DNA molecule and single ROI resolution. Reassuringly, both enrichment strategies again agree very well (Fig. 2I). Pearson correlation coefficients between experimental approaches (Open *r* = 0.59 (REST) and 0.78 (CTCF), nucleosome *r* = 0.79 (REST) and 0.83 (CTCF) and TF *r* = 0.85 (REST) and 0.92 (CTCF)) further corroborate the visual inspection of the data. In conclusion, NOMe-seq results in highly reproducible classification of chromatin states irrespective of the selected enrichment protocol, the respective experimenters, or even laboratories. Notably, when solely considering oligo synthesis costs (sgRNA oligos versus capture oligos), guidedNOMe-seq is more cost effective up to ~ 5000 ROI. For larger ROI numbers, designing a custom capture array would be more economical (Figure S1F). NOMe profiling, with and without Cas9-mediated enrichment of ROI has also been employed in combination with long-read Oxford Nanopore sequencing [19, 26, 27]. This method referred to as nanoNOMe-seq, produces much longer reads at the expense of throughput. Due to the long-read length, nanoNOMe is perfectly suited to study nucleosome positioning and TF binding spanning hundreds of nucleosomes for a handful of ROI while array-capture and guided-NOMe can only query 2–4 nucleosomes in a single sequencing read but for hundreds to thousands of ROI. Due to difference in studied organism (human/yeast vs mouse), we could not directly compare nanoNOMe with guidedNOMe inferred chromatin states.

We conclude that guidedNOMe-seq, array capture NOMe-seq and nanoNOMe-seq are nicely complementary both with their own strengths and weaknesses (see also Discussion).

### sgRNA design principles for guidedNOMe-seq

**Fig. 3:**
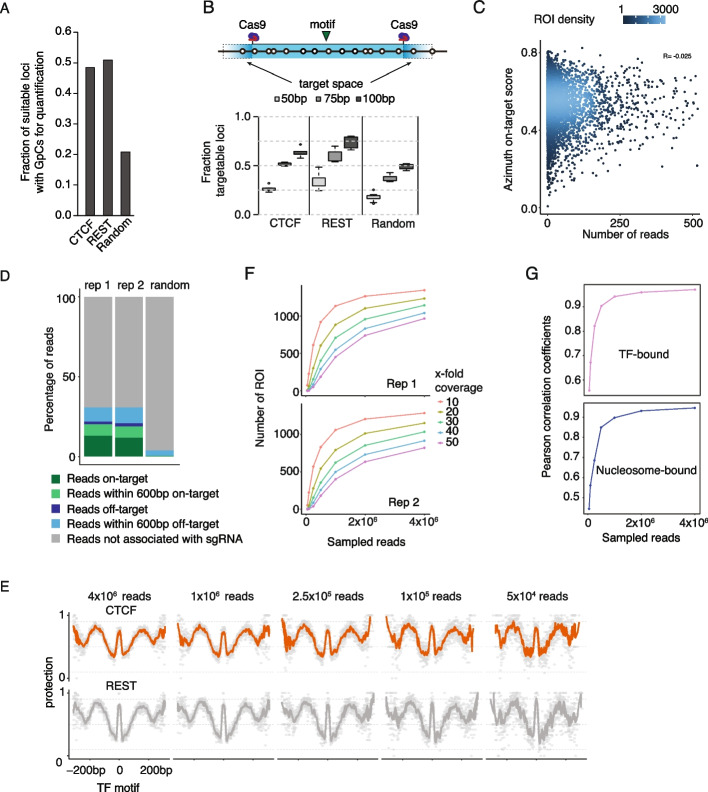
Strengths and limitations of guidedNOMe-seq. **A** Shows the fraction of Ctcf, Rest bound and Random genomic ROI that contain GpCs in all three chromatin state quantification windows. For the random genomic ROI we sampled 10,000 loci. **B** (top) Schematic representation of the in silico analysis (bottom) We randomly sampled 10 times 100 ROI and determined the fraction of ROI that can be targeted by Cas9 in every 100 ROI separately with three different sgRNA target spaces, as indicated. **C** Scatter plot showing the sgRNA on target scores predicted by the Azimuth algorithm versus the observed associated Cas9 cut site read start counts **D** Stacked barplot displaying the relationship between the sequenced reads and on- and off-target Cas9-sgRNA predictions. **E** ROI are split on TF and aligned on the center of the TF binding motif, as indicated. The y-axis shows the average protection a GpC position has against exogenous methylation by M.CviPI (1-mGpC). Loess line is added through the individual data points. We subsampled the reads, as indicated above the average plot (**F**) Line graphs depicting the read coverage over the ROI with increasing numbers of sampled reads in both replicates. **G** Shows the Pearson correlation score between replicates of the TF and nucleosome state classification with increasing numbers of sampled reads

As outlined above, the usability of ROI depends on the presence of at least one GpC in all three chromatin quantification windows (Fig. 1B). Therefore, we determined the fraction of ROI that fulfil this criterium. When bootstrapping random genomic regions, on average 20 percent of ROI in the mouse genome are suitable for quantification by guidedNOMe-seq. Focusing on ROI containing a REST or CTCF ChIP-seq peak and binding motif we found that approximately 50 percent of these regions are suitable (Fig. 3A). The observed higher percentage of suitable REST and CTCF associated ROI can be explained by the presence of GpCs in their binding motifs [28, 29]. The non-uniform distribution of GpCs throughout the genome and relative to a specific set of ROI is an inherent caveat of NOMe-seq that cannot easily be changed and hence needs to be taken into account when designing an experiment.

Further, the fraction of ROI that can be targeted depends on the stringency of sgRNA design parameters. Namely, the target space allowed for Cas9 surrounding the ROI and the chosen cutoffs regarding on- and off-target scores of the identified sgRNAs. In this study we used an 80 bp target space at 5’ and 3’ ends of each ROI, did not select for GpCs in the chromatin quantification windows because of the GC-rich binding motifs of the TFs of interest, neglected on-target scores and omitted sgRNAs with a very poor off-target score (see Methods).

To assess the impact of the above parameters, we performed in silico analyses with different settings. First, we investigated the relationship between Cas9 target space size and the number of targetable ROI for REST, CTCF and random genomic regions. Already with the smallest target space (50 bp) Cas9 can, depending on the analyzed ROI group, liberate between 1/5 -1/3 of the ROI. As expected, the fraction of targetable ROI increases when Cas9 target space increases. Notably, with a target space of 100 bp, Cas9 can target half or more of the ROI analyzed (Fig. 3B). Using these numbers, in combination with the GpC distribution analysis described above, we estimated that we can target between 9 and 26 k out of the 70 k CTCF ChIP-seq peaks depending on the chosen cut offs.

Thus, not surprisingly, increasing target space for sgRNA selection improves the number of targetable loci in the genome. Yet, this comes at the expense of correspondingly shortening the length of quantifiable DNA around the TF motif and must thus be considered carefully and adjusted to the read-length of the available NGS sequencing platform.

Next, we determined if in silico predicted sgRNA on- and off-target scores can be used to optimize sgRNA selection and read coverage uniformity of ROI. Surprisingly, this analysis making use of the 1500 ROI in our experimental set up revealed a very poor correlation between sgRNA on-target scores and observed read coverage over ROI. Towards this end, we employed seven different sgRNA prediction algorithms but none of them could predict cutting efficiency, respectively read coverage, satisfyingly (Figs. 3C and S2A) [30]. Nevertheless, we would still recommend in silico prediction of sgRNA on-target scores because our analyses revealed that sgRNAs with very poor predicted on-target scores and the presence of simple repeats in ROI should be avoided when possible (Figure S2B).

Lastly, we analyzed potential adverse effects of retaining sgRNAs with predicted off-targets. We first determined the percentage of reads associated with the on-target Cas9 cleavage activity. In both replicates we found ~ 10% of reads precisely originating from the predicted on-target sgRNA-Cas9 cleavage sites, but also noticed that another ~ 4% of reads were within 600 bp of the intended cleavage site (Fig. 3D and S2C). We attribute this to the combined effect of targeted digestion by Cas9 in combination with random breakage of genomic DNA on the other side, which stochastically results in DNA fragments of suitable sizes for NGS library generation. In addition, ~ 7 percent of reads are associated with predicted off-target sites, which is three times more than expected by chance (Fig. 3D). Consistent with literature, we found that predicted off-targets without PAM sequence contribute minimally to off-target reads (Figure S2D) [31].

In summary, the sgRNA analysis revealed that on-target score predictions vary considerably between used algorithms and correlate poorly with the observed ROI read count distribution. We therefore assume that ROI sequence features (e.g. simple repeats (Figure S2B)) and potential biases in the library preparation outweigh the selection of sgRNA on their predicted on-target score except for sgRNAs with a very low score. The sgRNA off-target analysis showed that using only perfect sgRNAs without off-targets will most probably decrease off-target sequencing reads. However, the gain will likely be minimal, as the majority (> 65%) of the reads not associated with a ROI are not linked to predicted sgRNA off-target sites but arise due to random genome breakage during the NOMe-seq protocol. Thus, careful handling of nuclei and isolated DNA, as recently optimized by Battaglia et al*.*, is an effective precaution to reduce off-target reads [19].

### Strengths and limitations of guidedNOME-seq

Despite the steadily decreasing costs for next-generation sequencing, financial constraints are still a major bottleneck for large experiments such as time-courses or comparative studies with dozens of samples. To determine the minimal sequencing depth that is required for reliable quantification of chromatin states by guidedNOMe-seq, we systematically down-sampled the guidedNOMe-seq libraries and subsequently performed analysis at different resolutions.

We first generated average profiles plotting the fraction of GpCs that are protected from methylation by M.CviPI per nucleotide, while keeping the CTCF and REST ROI separate. As expected, using 4 million guidedNOMe-seq reads, we observed M.CviPI protection footprints for the TF bound to its motif and for positioned nucleosomes up and downstream of the TF binding site (Fig. 3E). At this sequencing depth, almost all individual GpC protection values (grey dots) nicely align with the LOESS smoothed curve. When serially down sampling total read numbers, the average protection pattern indicated by the LOESS line persists throughout while the noise level of individual data points increases. However, even with as little as 50,000 reads, TF and nucleosome footprints were still detectable (Fig. 3E). To quantify the increase in single nucleotide noise we computed the Pearson correlation between the fraction of protection per nucleotide position with decreasing read numbers versus the full 4 million total reads sample. This revealed correlation coefficients ranging from 0.92–0.64, indicating that with as little as 50 k reads, reproducibility is still very high (Figure S2E).

We next focused our analysis on the chromatin state quantification of single ROI by assessing the GpC protection state of individual DNA molecules (according to Figs. 1B and 2F). We followed a similar down-sampling approach as described above to determine how chromatin state classifications perform under limited coverage conditions. In contrast to the average plots, the accuracy of the individual ROI chromatin state classification rapidly deteriorates with decreasing read numbers (Figure S2F). This is likely caused by an increase in sampling noise due to the lower coverage per ROI at lower sequencing depths (Fig. 3F). To determine the required sequencing depth, we compared Pearson correlation coefficients of inferred ROI chromatin states (TF-bound and Nucleosome-bound) between replicates with increasing subsampled read counts. In line with the replicate scatterplots for the TF chromatin state, this showed a steep increase in correlation coefficient with increasing read numbers, which starts to flatten out between 1 and 4 million reads. That is, sequencing more than 4 million reads for 1500 ROI can increase reproducibility, albeit only relatively small improvements are likely (Fig. 3G).

In summary, we establish the experimental boundaries of guidedNOMe-seq and find that biological reproducibility of the protocol is excellent, without applying any normalization steps. Hence, guidedNOMe-seq is an important extension to existing NOMe-seq protocols, enabling researchers to quantify how perturbations influence chromatin states on hundreds of loci in parallel.

### guidedNOMe-seq reveals asymmetric nucleosome patterns at ChAHP-bound loci

**Fig. 4:**
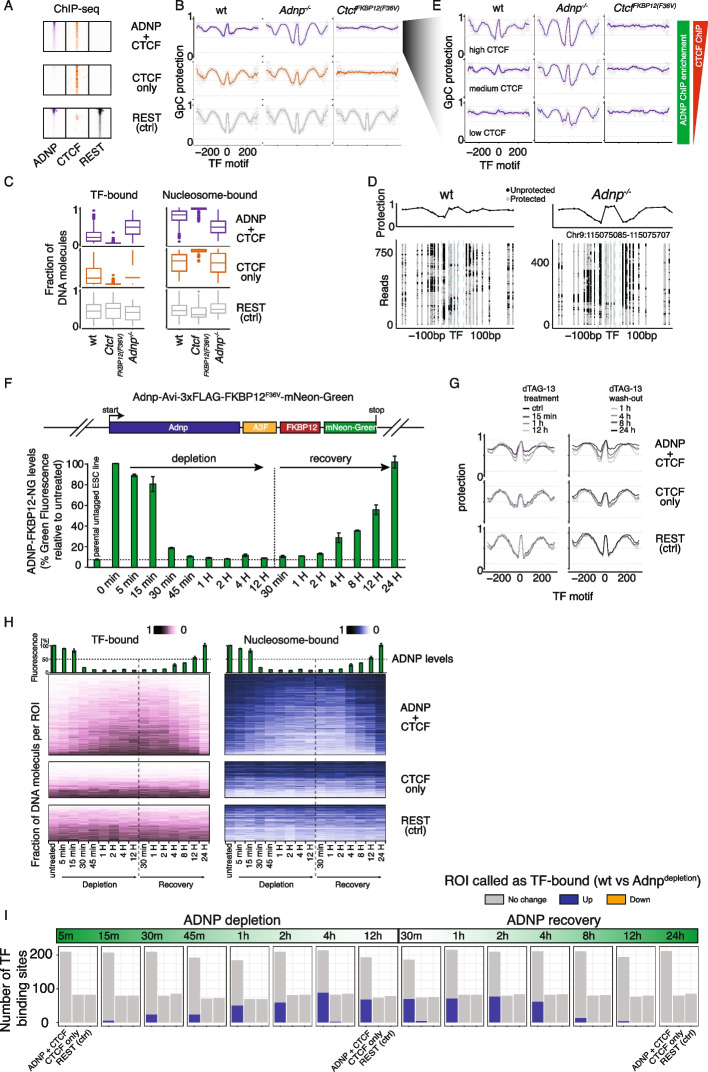
guidedNOMe-seq quantifies chromatin state changes upon ChAHP perturbation. **A** Heatmaps displaying ChIP-seq signal for ADNP, CTCF and REST in the three ROI groups, as indicated. **B** ROI are split on TF (REST and CTCF) and the CTCF group is further divided on its relation to Adnp, as indicated. ROI are aligned on the center of the TF binding motif, as indicated. The y-axis shows the average protection a DNA position has against exogenous methylation by M.CviPI (1-mGpC). Loess line is added through the individual data points. Average plots were generated under wild type conditions, after 24H Ctcf depletion, and in the absence of Adnp, as indicated. **C** Boxplots summarizing the observed fraction of TF and nucleosome chromatin states in the ROI under wild type, Ctcf depletion and Adnp loss conditions, as indicated. **D** Example of a single ROI where CTCF co-binds with Adnp and its surrounding (~ 250 bp ±). Top plot shows the average protection value of the GpCs at the indicated location. Bottom plot shows protected and unprotected state of the GpCs in individual DNA molecules. Single ROI plots were generated under wild type conditions and in the absence of Adnp, as indicated. **E** Ctcf ROI co-bound by Adnp are split on Ctcf levels as observed by ChIP-seq, as indicated on the right. ROI are aligned on the center of the TF binding motif, as indicated. The y-axis shows the average protection a DNA position has against exogenous methylation by M.CviPI (1-mGpC). Loess line is added through the individual data points. Average plots were generated under wild type conditions, after 24H Ctcf depletion, and in the absence of Adnp, as indicated. **F** (top) A schematic overview of the TALENs mediated genome engineering performed at the endogenous Adnp locus. (bottom) FACS analysis of the dTAG-13 treatment and recovery time course. On the y-axis the time after dTAG-13 addition or dTAG-13 wash-out is indicated. On the x-axis the measured green fluorescence. **G** ROI are split on TF (REST and CTCF) and the CTCF group is further divided on its relation to Adnp. ROI are aligned on the center of the TF binding motif, as indicated. The y-axis shows the average protection a DNA position has against exogenous methylation by M.CviPI (1-mGpC). Loess line is added through the individual data points. Average plots off 8 selected timepoints from the Adnp depletion and recovery time course. **H** (left) Heatmap displaying the TF chromatin state dynamics of all individual ROI throughout the Adnp depletion and recovery timecourse. (right) Heatmap displaying all nucleosome chromatin state dynamics of the individual ROI throughout the Adnp depletion and recovery timecourse. **I** Stacked barplot showing the three different ROI groups and the amount and direction of ROI that display significantly changes in their TF chromatin states as determined by DESeq2 throughout the Adnp depletion and recovery time course

To exemplify the power of guidedNOMe-seq to quantitatively measure how chromatin states change upon perturbations, we used genetic inactivation of the ChAHP complex as a case study. We have previously shown that the ChAHP complex, consisting of the chromatin remodeler CHD4, the zinc-finger protein ADNP, and heterochromatin protein HP1gamma, binds over 15,000 loci in mESCs [32]. These ChAHP-bound loci often reside in a repetitive SINE B2 element and are also enriched for CTCF. When ChAHP function is perturbed (e.g. through the removal of ADNP), chromatin accessibility and CTCF binding increases specifically at ChAHP-bound sites, suggesting that ChAHP competes with CTCF for binding on chromatin and/or restricts chromatin accessibility at those sites [32, 33]. However, the molecular mechanisms behind ChAHP function and its interplay with CTCF are not well understood. We thus set out to quantify nucleosome occupancy and TF binding in *Adnp*^*−/−*^ mESCs and compared this to the guidedNOMe-seq data set from wild type and CTCF-depleted mESCs. To address the interplay between ADNP/ChAHP and CTCF, we subdivided the CTCF ROI into two groups: group 1 contains ROI where both ADNP/ChAHP and CTCF are enriched and group 2 consists of ROI where only CTCF is bound based on ChIP-seq data (Fig. 4A) [33]. Reassuringly, the protection profile in the REST-bound control group is very similar between all conditions, further validating the high reproducibility of guidedNOMe-seq between experiments and conditions (Fig. 4B). At loci where both CTCF and ADNP/ChAHP are enriched, we find a pronounced increase of positioned nucleosome upon ADNP loss. Loci only bound by CTCF do not show a different protection/nucleosome positioning pattern in the absence of ADNP/ChAHP. In contrast, the protection pattern of CTCF-associated groups is markedly changed following 24 h of CTCF depletion. Here, the characteristic GpC protection pattern in wild type cells is lost. Of note, the GpC protection after CTCF depletion is at a similar level as the protection elicited by + 1/-1 nucleosomes in wild type cells, indicating that the ROI are not in an open, nucleosome free state when CTCF is depleted, but rather occupied by non-positioned nucleosomes (Fig. 4B). This random nucleosome positioning is supported when inspecting individual ROI where we observe large (> 100 bp), seemingly randomly located stretches of protected GpCs in individual DNA molecules (Figure S3A). When quantified at the single ROI level, we find that around 20% of DNA molecules are TF bound in the two CTCF-associated groups at any given moment in wild type cells. Specifically, in the CTCF group associated with ADNP/ChAHP, this increases to ~ 50% in the absence of ADNP, whereas upon CTCF depletion TF binding decreases to nearly 0 percent in both CTCF-associated groups (Fig. 4C).

To determine which ROI show significant changes in their chromatin state, we used an implementation of edgeR in our dinoR package. This revealed that indeed many ROI undergo a statistically significant chromatin state change from nucleosome bound to TF-bound between wild type and Adnp KO samples (Figure S3B-E). Accordingly, the increase in TF-bound chromatin states in *Adnp*^*−/−*^ cells is coupled to a decrease in nucleosome bound DNA molecules and a small increase in open chromatin. Further, these results indicate that the nucleosome and TF protection patterns observed in wild type cells are largely dependent on CTCF, as positioning is nearly completely lost upon CTCF depletion.

Of note, when comparing the wild type protection patterns between CTCF bound and CTCF and ADNP/ChAHP co-bound loci, we noticed an asymmetry in the ADNP-bound group in some single ROI plots which we never observed in the CTCF or the REST single ROI plots (Fig. 4D). To further explore this, we split this group of ROI based on CTCF ChIP enrichment (high – medium – low) and generated average protection profiles (Fig. 4E). The asymmetry is primarily visible in the two groups with medium and low CTCF levels. Interestingly, the maximum GpC protection is similarly high on both sides of the binding motif, indicating that the fuzzier downstream region is occupied by nucleosomes, which however are not well-positioned in contrast to the upstream region. Upon *Adnp* loss the asymmetry is lost in all groups, indicating that the fuzzy nucleosome positioning downstream of the binding site might indeed be due to ChAHP. Hence we wanted to quantify the observed asymmetry with single ROI resolution. To do this we adapted the chromatin quantification window approach and introduced two novel states, namely “upstream positioned nucleosome” and “downstream positioned nucleosome” (Figure S4A). Analyzing these two additional chromatin states revealed that ~ 30% of the ADNP/CTCF co-bound ROI significantly lose DNA molecules classified as positioned nucleosome upstream in the absence of *Adnp* (Figure S4B and C). At present we do not know how the observed asymmetry is established and if it has a functional role in reducing CTCF binding. But this likely results from the chromatin remodeling activity of CHD4, which has previously been shown to reduce access to chromatin by increasing nucleosome densities in a non-positioned manner at its target loci [34–37]. These data confirm that CTCF only and REST-bound sites show symmetric nucleosome positioning, whereas CTCF and ADNP enriched ROI show asymmetry in their nucleosome positioning that correlates with the presence of ChAHP.

### guidedNOMe-seq reveals insights into the interplay between ChAHP and CTCF with temporal resolution

Most of our knowledge regarding the function of genes in biological systems is through the generation of constitutive gene knock-out models and subsequent observation of loss-of-function phenotypes. These studies have proven very powerful, but measure endpoint situations without temporal resolution and can therefore be prone to secondary effects. With the recent development of small-molecule inducible degradation tags that allow rapid and specific degradation of endogenously tagged proteins, high-resolution time course measurements of biological processes became feasible [38, 39]. Such experiments have for example been performed to measure the temporal dependency of 3D genome organization on the presence of the cohesin subunit RAD21 [40]. Also, it proved to be a powerful approach to assess the relevance of chromatin remodelers like BRG1 for TF binding to DNA [40, 41].

Unfortunately, high resolution time course experiments coupled to genome-wide read-outs such as ChIP-seq, HiC, ATAC-seq, or RNA-seq are still very expensive and often require complex normalization procedures for data analysis due to sparsity of data-points. As described above, guidedNOMe-seq requires relatively low sequencing depth to achieve excellent reproducibility without any normalization and therefore appears to be ideally suited to measure chromatin state dynamics during time course experiments.

To test if guidedNOMe-seq can provide insight into chromatin state dynamics, we established an endogenous gene fusion of *Adnp* with FKBP12^F36V^ and mNeon-Green (Fig. 4F; top). The FKBP12^F36V^ tag allows for rapid degradation of ADNP upon addition of the dTAG-13 compound, whereas mNeon-Green allows for the direct quantification of ADNP protein levels by flow cytometry [38]. Furthermore, the FKBP12^F36V^ degradation system is reversible. Upon dTAG-13 wash-out, ADNP levels should slowly restore, making it also possible to perform Adnp recovery time-course experiments as well. We first established the depletion and recovery kinetics of ADNP after dTAG-13 treatment and wash-out (Fig. 4F; bottom). In agreement with studies on other proteins, we found that the FKBP12^F36V^ tag leads to rapid and near complete depletion of ADNP upon addition of dTAG-13 [38, 41]. 5 min after dTAG-13 addition, a small decrease in mNeon-Green signal was already observable. After 1 h of treatment, fluorescence intensity was close to background levels (untagged control). Recovery of protein levels after dTAG-13 wash-out is largely dependent on protein synthesis rates. In the case of Adnp recovery started 2 h after wash-out and steadily increased to about 35% after 8 h and recovery was complete 24 h after wash-out. We next generated guidedNOMe-seq libraries for these 16 time points in biological duplicates throughout the ADNP depletion and recovery time course. Utilizing the same three ROI classes as before, the GpC protection pattern changed as early as 15 min after initiation of ADNP depletion specifically at ROI where ADNP is present (Fig. 4G). The methylation protection at the CTCF motif and the positioning of nucleosomes steadily increased during the first hour of ADNP depletion and was comparable to that of *Adnp*^*−/−*^ cells at later time points (Fig. 4B and G). Upon reversal of the depletion (dTAG-13 wash-out), we observed first signs of recovery towards the untreated state after 8 h. Reversion was completed within 24 h. These results show that ChAHP bound ROI are converted into CTCF bound sites upon ADNP depletion within an hour. In the other direction, although the reversal of CTCF bound sites to ChAHP controlled loci is constrained by ADNP protein synthesis rates, we observe the first signs of the reversal 8 h after dTAG-13 wash-out when ADNP protein levels are back to 35% compared to untreated cells (Fig. 4F).

We next set out to quantify chromatin state dynamics, as introduced in Fig. 1B, with single DNA molecule resolution throughout the time course. The ROI present in the ADNP/CTCF group showed a marked increase in TF-bound chromatin state already after 15–30 min of Adnp depletion while the number of nucleosome-bound molecules reduced correspondingly (Fig. 4H and I). On the other hand, CTCF only and the REST control group showed minimal dynamics throughout the time-course, in line with the above average analysis. To statistically stratify these observations dinoR was used. This revealed that after 15 min of ADNP depletion we already find the first ROI to show a significant increase in TF chromatin state. The number of significant changes increases during the first hour and then remains constant thereafter. Intriguingly, this increase in TF chromatin states is completely reverted 24H after dTAG wash-out (Fig. 4I).

Of note, the ADNP associated asymmetric nucleosome positioning that we identified in wild type vs *Adnp*^*−/−*^ ESCs (Fig. 4D, E and S4B, C), follows ADNP levels throughout the depletion and recovery time course, which again supports the idea that the downstream loss of precise nucleosome positioning is due to the presence of functional ChAHP complex (Figure S4D). However, further experiments will be required to unequivocally attribute this effect to a specific activity of ChAHP and to investigate its consequence for CTCF binding.

These data demonstrate that the ChAHP complex is continuously required to counteract CTCF binding at its target sites. The ADNP recovery experiments show that there are no signs of long-term epigenetic memory that would reduce ChAHP activity at CTCF loci. This strongly suggests that the ChAHP-CTCF binding landscape is established cell autonomously in a rapid and reversible manner.

Taken together, we conclude that guidedNOMe-seq is a powerful technique to quantify how genetic or small-molecule induced perturbations influence the chromatin state at hundreds of individual ROI either at steady-state conditions or in time-course series with many samples requiring minimal amounts of NGS sequencing.

## Discussion

Despite its many advantages, NOMe-seq is currently not widely used [14, 16, 18, 19, 27, 42–45]. We hope that this study and the extension of the NOMe-seq tool kit presented here will help to position guidedNOMe-seq as a cost-efficient, highly accurate alternative to whole-genome or microscopy based single cell/molecule methods. The protocol can be completed within two days with standard molecular biology laboratory equipment omitting the requirements for highly specialized set-ups. We show that NOMe-seq based experiments result in very reproducible data even when executed in different laboratories, without the need for complex high-dimensional analysis, nor any normalization steps. Furthermore, analyzing our internal control (the REST bound ROI) shows that guidedNOMe-seq has a false positive rate of only 0.7% (Fig. 4I and Methods). As with all methods, also NOMe-seq has inherent limitations. For instance, not every TF is equally suitable for NOMe profiling. Previous work by Sönmezer et al. showed that REST and CTCF bound sites are suitable for NOMe profiling, whereas other TFs (e.g. SOX2 and STAT2) show weaker signals. This is likely caused by different binding modes/affinities, resident times and/or TF abundance within the cell. Another limitation is the need for naturally occurring GpCs (Fig. 3A). Krebs et al*.* mitigated this problem by performing foot printing using two methyltransferases that target cytosines present in two different (GpC and CpG) sequence contexts, but this only works in organisms without endogenous CpG methylation, or in cells that allow the deletion of the endogenous DNA methylation machinery [42, 46]. A promising development has been the recent identification and characterization of three cytosine methyl transferases (MTase) with complementary sequence context specificities (TCTG, CC and CNG) [47]. Combining these three MTases with the GpC MTase M.CviPI could thus significantly increase the median resolution and the number of genomic loci amendable to NOMe-seq.

Another major hurdle towards utilizing current single-cell/allele methods are the complex bioinformatic analysis required once the data are collected. Since NOMe-seq is a normalization-free direct read out of chromatin occupancy of TFs and nucleosomes, it is much simpler to analyze. In order to also facilitate this step and to enable more researchers to employ NOMe-seq approaches, we have developed an R-package called **di**fferential **NO**Me (dinoR). dinoR allows easy visualization and quantification of individual NOMe-seq samples, as well as the statistical stratification of chromatin state differences between two conditions. This analysis framework utilizes a standardized data structure suitable for any type of NGS data of bisulfite converted DNA and therefore allows evaluation of whole genome, array capture and guided NOMe-seq data and ultimately should facilitate using different R-packages without the need to change data structure. Together, this makes the elegant NOMe-seq technology variants accessible to a wide range of experimental biologists and helps to establish stringent data analysis standards for reproducibility and exchange across laboratories.

In this manuscript we present guidedNOMe-seq and compared it to array capture NOMe-seq, which both use short read sequencing to infer local chromatin states [14]. Another related approach uses Cas9 enrichment of NOMe treated DNA followed by long read sequencing (nanoNOME-seq) [19]. Here we will briefly recap the results section and further discuss the pros and cons of these three approaches.

### guidedNOMe-seq vs array capture NOMe-seq

For an experimental set up that primarily focuses on TSSs and common/predicted cis-regulatory elements (CREs) in human or mouse, the array capture NOMe-seq protocol is the method of choice. When working with different species and/or when the focus is rather a specific transcription factor with binding sites distal to TSSs and predicted CREs or repetitive regions, guidedNOMe-seq is the preferred method because it allows investigation of thousands of custom defined ROI without relying on commercially available tools.

### Short read NOMe-seq vs long read nanoNOMe-seq

When performing nanoNOMe-seq long (~ 100 kb) DNA molecules are sequenced [19]. This allows interrogation of individual TF binding sites, the nucleosome positioning in their flanking sequences and even the chromatin state relationship between more distal elements. This makes nanoNOMe a very versatile and powerful approach for studying individual loci to in detail while in turn suffering from the following limitations: 1) the scale at which nanoNOMe has so far been executed and currently is feasible in a cost-efficient manner is almost two orders of magnitude smaller as compared to guidedNOMe-seq or array capture NOMe-seq (10–30 vs >  = 1500 ROI). (2) nanoNOMe requires large quantities of input DNA precluding the analysis for low input samples. (3) The obtained on-target percentage of nanoNOMe is approximately 40 times lower as compared to guidedNOMe-seq when keeping the tenfold lower read counts obtained when performing Cas9 enrichment followed by nanopore sequencing in mind [22].

Given this, we conclude that, at present, nanoNOMe is the technique to interrogate the relationship of chromatin states between distal elements within a single DNA molecule with low ROI throughput. In contrast, when the interest is to quantify local chromatin states at near genome-scale, array-capture NOMe or guidedNOMe are the preferred methods of choice.

## Conclusions

The field of genomics has experienced the emergence of a plethora of high resolution and single-cell/allele methods that are pivotal for a more quantitative analysis of the molecular mechanisms underlying genome regulation. Most of the protocols available to date either require highly customized set ups, such as for high-resolution and single-molecule microscopy, or very high read depth, in the case of next-generation sequencing based techniques, to achieve the necessary sensitivity for single-locus quantification. Moreover, single-cell omics approaches are expensive and challenging to analyze and reproduce because of the inherent high levels of technical noise in the data. NOMe-seq coupled with array capture, or Cas9-mediated target enrichment described here, can make up for several of these shortcomings. This however comes at the expense of throughput and the need to select ROI. Nevertheless, it is feasible to acquire quantitative data for hundreds to thousands of individual loci at single allele resolution in one experiment, including multiple conditions, perturbations, and replicates. In our study we used a pool of 6000 sgRNAs to perform target enrichment. Recently, depletion experiments were successfully done with a pool of ~ 500 000 sgRNAs in a single Cas9 digestion experiment [48]. This indicates that the number of sgRNAs and therefore ROI can be significantly increased for guided-NOMe-seq as well*.*

## Methods

### Wetlab

#### Oligo pool amplification

The sgRNA oligo pool (see supplemental tables) was ordered at Twist Bioscience and amplified according to their recommendations with some minor modifications. Briefly, the oligo pool was dissolved in 10 mM Tris buffer, pH8 to a concentration of 5 ng/ul. We used the recommended KAPA HiFI PCR kit (Roche Cat # KK502) according to the manufacturer instruction. PCR mix was as follows: 12.5 ul KAPA HiFI HotStart Ready Mix, 0.75ul 10uM Fwd Primer, 0.75ul 10uM Rev Primer, 2ul oligo pool (5 ng/ul) and 9ul H2O (see Table 1 for primer sequences used). We performed PCR with the recommended cycling protocol: using 56 °C annealing temperature, 15 s extension and performed 16 cycles of amplification. PCR product was purified using 1.8 V Ampure beads (Beckman) and resuspended in 25ul of H2O. DNA concentration was measured using the qubit dsDNA HS kit (Thermo Fisher Scientific) and correct size was confirmed once by running an appropriate PCR aliquot on a High Sensitivity DNA bioanalyzer chip (Agilent).

**Table 1:** Oligos used for genome editing and PCRs

**NOMe PCR Amplicons**	**strand**	**sequence**
chr4:135534271-135535026	Fwd_1	ttgtttttataatattaggtttagggt
	Fwd_2	tagggttatagatttatatttgtgg
	Rev_1	AaACTCAACCCCACCACAaATATCTCAA
	Rev_2	aTCTTTAAaAaTAAaAAaaTTTCTaAaAAC
chr5:103691073-103691705	Fwd_1	AtAtTTTtTGGATtTTATtTTtAATAtAAtt
	Rev_1	ACAAAAaTCACTTACTTAaATAAACTATCCAaTa
	Rev_2	CTATCCAaTaTCTAaaaAAAAaAaTATAATa
chr5:113963199-113963929	Fwd_1	GGTtAAAATTTAGGAAAAtAGAtAGAGTt
	Fwd_2	GGGGAGTtttAGGTAGAATATGAGTtTGG
	Rev_1	TTTaTTTCTCCTaaaTCCCTCTaTCCCTaTaaT
	Rev_2	CAaTaTCTCCCTATTaAaACCCTaT
chr8:64676254-64677021	Fwd_1	GTTTtAtTGAtAAAATGAAAATtTGAtAGTG
	Fwd_2	ttAAAtTGGGGAtAAAtAGGTtAATATG
	Rev_1	taaaattcataaaaatccacctacttca
	Rev_2	ttcctaaatactaaaattaaaaatat
chr8:123132736-123133458	Fwd_1	tttttgagtgttgggaatttaagtgtga
	Fwd_2	tTGTATGTAtgtagatggttgtga
	Rev_1	tcttacaaaaaaccaaaatttaattctca
chr9:118708066-118708565	Fwd_1	TGTtTGTtTATATGGTTAAAGAAAATTAG
	Fwd_2	GTTAtTTTTAGTAAGTTTATTAAAATTGAG
	Rev_1	aTCTaAAaAATTTCCCCAAAaTCAaAa
chr10:81577506-81578424	Fwd_1	Atttatttaattttatatgtatgggtgttttg
	Fwd_2	atgtatgggtgttttgtttatatatttgt
	Rev_1	aaaatatctcaataaataaaaatatt
chr11:19860509-19861184	Fwd_1	ttAAGGAtttTAttTGTTTttAgtagtgg
	Fwd_2	tgtaattttattagttaggaggttga
	Rev_1	cattcttaattaaaattcttaactaAAaaTaaa
chr13:37461486-37462258	Fwd_1	gggagattttattttatttaaaataGTAT
	Fwd_2	AtTtTTTTtttTAtAAAttAAAGTTttAAGAA
	Rev_1	aATaaAAAACAACAACAACAACAAAAATTaTC
	Rev_2	AaTAaACATaaTAaaTAATAaaAaaACACATC
chr18:34114859-34115360	Fwd_1	GTTGGAATttTGAttAAGAAGAGTATG
	Fwd_2	GATTTTtAtAAATAATTttAtAtAtTTAAGGG
	Rev_1	CaTTTTAaAAAACACAACAAAAACCATAACA
	Rev_2	TAATTACCTTTaaaATaTTTAAaTCTTAA
Chr15:97952351:97952777	Fwd_1	TGTTATGTTTTTGGGATTTATTGAAT
	Rev_1	CCTTTCCCAATCCTAAACTAACT
Chr2:32912400:32912825	Fwd_1	ATTGGGGGTTTATGGGATTAATAG
	Rev_1	TATTCACATCTCTCTACAAAAACCA
chr8:70873668:70874090	Fwd_1	TGTTTAATTATTTTTGAGATAGTGTT
	Rev_1	CCATCTCAAATATTAATAAACAAAAA
chr11:62756820:62757164	Fwd_1	ATGAAGAGTTTTTGAATGAAGGTTAA
	Rev_1	CTTAAATTATCCACCCTCCTCTCTAA
chr4:130138383:130138741	Fwd_1	AGGGAGATATTAAAGATTTAGGTTT
	Rev_1	CATCCTCTTAACACCAATCTAAAAT
**oligos for generating gRNA pools**	**strand**	**sequence**
to amplify guidedNOMe libraries from ends of p5 and p7 (this is P5)	fwd	AATGATACGGCGACCACCGAGAT
to amplify guidedNOMe libraries from ends of p5 and p7 (this is P7)	rev	CAAGCAGAAGACGGCATACGAGA
Fwd In vitro sgRNA Twist Oligo pool amplification	fwd	aagcTAATACGACTCACTATAGG
Rev In vitro sgRNA Twist Oligo pool amplification	rev	AAAAGCACCGACTCGGTG
**oligos for genome editing**	**strand**	**sequence**
Adnp-avi-FLAG-FKBP12-Neongreen HR donor gBLOCK		aaaGGCTACAGTGCAAGATGACACAGAGCAGTTAAAATGGAAGAATAGTTCCTATGGAAAAGTTGAAGGGTTTTGGTCCAAGGACCAGTCACAGTGGGAAAATGCATCTGAGAATGCAGAGCGCTTACCAAACCCACAGATTGAGTGGCAGAATAGCACAATTGACAGTGAGGACGGGGAGCAGTTTGACAGCATGACTGACGGAGTTGCTGATCCCATGCATGGCAGCTTAACTGGAGTGAAGCTGAGCAGCCAGCAAGCCcctggtGGCCTGAACGACATCTTCGAGGCTCAGAAAATCGAATGGCACGAAgctgactataaggaccacgacggagactacaaggatcatgatattgattacaaagacgatgacgataagGGAGTGCAGGTGGAAACCATCTCCCCAGGAGACGGGCGCACCTTCCCCAAGCGCGGCCAGACCTGCGTGGTGCACTACACCGGGATGCTTGAAGATGGAAAGAAAGTTGATTCCTCCCGGGACAGAAACAAGCCCTTTAAGTTTATGCTAGGCAAGCAGGAGGTGATCCGAGGCTGGGAAGAAGGGGTTGCCCAGATGAGTGTGGGTCAGAGAGCCAAACTGACTATATCTCCAGATTATGCCTATGGTGCCACTGGGCACCCAGGCATCATCCCACCACATGCCACTCTCGTCTTCGATGTGGAGCTTCTAAAACTGGAAATGGTCTCCAAGGGCGAGGAGGATAACATGGCCTCTCTCCCAGCGACACATGAGTTACACATCTTTGGCTCCATCAACGGTGTGGACTTTGACATGGTGGGTCAGGGCACCGGCAATCCAAATGATGGTTATGAGGAGTTAAACCTGAAGTCCACCAAGGGTGACCTCCAGTTCTCCCCCTGGATTCTGGTCCCTCATATCGGGTATGGCTTCCATCAGTACCTGCCCTACCCTGACGGGATGTCGCCTTTCCAGGCCGCCATGGTAGATGGCTCCGGATACCAAGTCCATCGCACAATGCAGTTTGAAGATGGTGCCTCCCTTACTGTTAACTACCGCTACACCTACGAGGGAAGCCACATCAAAGGAGAGGCCCAGGTGAAGGGGACTGGTTTCCCTGCTGACGGTCCTGTGATGACCAACTCGCTGACCGCTGCGGACTGGTGCAGGTCGAAGAAGACTTACCCCAACGACAAAACCATCATCAGTACCTTTAAGTGGAGTTACACCACTGGAAATGGCAAGCGCTACCGGAGCACTGCGCGGACCACCTACACCTTTGCCAAGCCAATGGCGGCTAACTATCTGAAGAACCAGCCGATGTACGTGTTCCGTAAGACGGAGCTCAAGCACTCCAAGACCGAGCTCAACTTCAAGGAGTGGCAAAAGGCCTTTACCGATGTGATGGGCATGGACGAGCTGTACAAATGAGGCCCTGGCGTGCCATAGCATATGCATATGGGCCGTGTTGCATCCTGGACTTCTGCTCTCCTTCCAGTCTGACTGCAAAGCTGTCTTCTAACTGGCACTACCTTGCAAGGACTGGTCAGTCAGCAGGCTGTGGGGATGTGTGACCACTGTAGTCTCAGTGGTTATTTCCAAGTCTATGATAGATGACTGGTTGATCTTTGTTCAGACTCT
Adnp 3' tagging genotyping primer FWD	fwd	GGTCCAAGGACCAGTCACAG
Adnp 3' tagging genotyping primer REV	rev	CTGACCAGTCCTTGCAAGGT
Ctcf-FKBP12-2xHA HR donor gBLOCK		TGTCAGTATTTGGTATCTGACAATTTCTAGCCTTTTGGGATTTTATGTGTGGCCACTTAACGTTCGCAGGGCTGTTTTGTTTCTGCTGACTTGGGCATCACTGCTGAGGCTTTCTTGTTGCTGCATCCCATTCATTGTCAGCATCGGGAACAATGCCTGTGCTCGCTGGGGGCTTTAATGTACGTACCCTTTGTTTTGTTCCTGCCCTTCTTTGCCAGCAACAGCCATCATTCAGGTCGAAGATCAGAATACAGGTGCAATTGAGAACATTATAGTTGAAGTCAAAAAGGAGCCAGATGCCGAGCCTGCGGAGGGGGAAGAAGAGGAGGCTCAGGCAGCCACCACAGACGCCCCCAACGGAGACCTCACGCCTGAGATGATCCTCAGCATGATGGACCGGGGGGGAGCAGGAGGCGTCGACGGTATCGATGGAGTGCAGGTGGAAACCATCTCCCCAGGAGACGGGCGCACCTTCCCCAAGCGCGGCCAGACCTGCGTGGTGCACTACACCGGGATGCTTGAAGATGGAAAGAAAGTTGATTCCTCCCGGGACAGAAACAAGCCCTTTAAGTTTATGCTAGGCAAGCAGGAGGTGATCCGAGGCTGGGAAGAAGGGGTTGCCCAGATGAGTGTGGGTCAGAGAGCCAAACTGACTATATCTCCAGATTATGCCTATGGTGCCACTGGGCACCCAGGCATCATCCCACCACATGCCACTCTCGTCTTCGATGTGGAGCTTCTAAAACTGGAAggcggctacccctacgacgtgcccgactacgccggctatccgtatgatgtcccggactatgcaggctccggaTAATGATGCTGGGGCCTTGCTCGGCACCAGGACTATTGGGCTGTGTTTAAACGGCCCAAATCTTAAtttttctcttttttttCTTTGGCTTTGGGAACGGCATAATTTTACACCATTTTACCAAACATACTGAGAACGAAAACTTCAAGGATGATGTTAGAAAAATGTGATTTAACTAGAACTTGTTTGATGTTAGCAAATCATGGAATGTTCTAAGTCTCTGAGGGTTTACTGTGAAGTGTTGAGGACAGTGTTGATGCCTAACTAGTTTTCTTAGATGGAAACAGAGACATTGAGCCCTCTCTCTTGATCGTAAACCACTCCAGAACGGCCACGGGTTTCCCAGAGTTCTATGGTCTTCCCAAGAGAATTTTTAATTGTAAATGCAGACTTGGGAAGGACT
Ctcf genotyping	fwd	GCATGCCATCCTACTGGTGTGC
Ctcf genotyping	rev	GCATGCCATCCTACTGGTGTGC
Ctcf gRNA oligo	fwd	aaacCAGCATGATGGACCGGTGATc
Ctcf gRNA oligo	rev	caccgATCACCGGTCCATCATGCTG
Adnplys407 mutation HR donor gBLOCK		GATAGCTCCCAAACCTCAAGACAAAAAGGGCATGGGACTCCCACCACGAATCAGCTCCCTTGCTTCTGGAAATGTCCGGTCGTTGCCATCACAGCAGATGGTAAACCGATTGTCAATACCAAAGCCCAACTTAAATTCAACGGGAGTCAACATGATGTCCAATGTTCACCTGCAGCAAAACAACTATGGAGTCAAATCTGTGGGCCAGAGCTATGGTGTTGGCCAGTCAGTGAGGCTGGGACTAGGTGGCAATGCTCCAGTTTCCATCCCTCAACAGTCTCAGTCCGTGAAACAGTTACTTCCAAGTGGGAATGGGAGGTCTTTTGGGCTAGGTGCTGAGCAGAGGCCCCCAGCAGCAGCCAGGTACTCCCTGCAGACTGCCAACACCTCTCTACCaCCAGGCCAAGTGGTCTCCCTCTGTGTCTCAGTCACAGGCATCgactataaggaccacgacggagactacaaggatcatgatattgattacaaagacgatgacgataagGGGAAGCCTATCCCTAACCCTCTCCTCGGCCTCGACTCGACGTAGAGTATTAGGTCAGTCCAGTTCTAAACCTCCACCAGCCGCCACAGGCCCTCCTCCAAGCAACCACTGTGCCACTCAGAAGTGGAAAATCTGTACAATCTGTAACGAGCTTTTCCCTGAGAATGTCTATAGCGTTCACTTCGAAAAGGAGCATAAAGCTGAGAAAGTCCCAGCCGTAGCTAACTACATTATGAAAATACACAATTTTACTAGCAAATGCCTCTACTGTAATCGCTATTTGCCTACAGATACCCTACTCAACCATATGTTAATTCATGGTCTGTCTTGTCCGTATTGCCGTTCCACCTTCAATGATGTAGAGAAGATGGCAGCACACATGCGAATGGT
Adnplys407 genotyping	fwd	GAGGACCATGAACGGATAGG
Adnplys407 genotyping	rev	ACTTTTGGTTGTGGCTTTGG
Adnplys407 gRNA oligo	fwd	caccgGGGAGACTTCACTTGGCCTG
Adnplys407 gRNA oligo	rev	aaacCAGGCCAAGTGAAGTCTCCCc

#### NOMe treatment

NOMe was performed essentially as described in Grand et al*.* [16] with two minor modifications. (1) After counting the cells, we processed ~ 0.8 × 10^6 cells throughout the initial lysis and washing steps. After washing, the nuclei pellet was resuspended in 189ul 1 × M.CviPI buffer, resuspended and 94.5 ul (0.4*10^6) cells was used for M.CviPI profiling. (2) Lysis of the cells to obtain nuclei was performed for 7.5 min (instead of 10 min) on ice.

#### In vitro* sgRNA transcription*

For in vitro sgRNA synthesis the EnGEN sgRNA synthesis kit (NEB) was used according to manufacturing’s recommendation, with minor modifications. We used 1uM (82 ng) PCR product of the amplified oligo pool and in vitro sgRNA reaction was performed for 2 h @ 37 °C to increase yield. After DNAseI treatment in vitro transcribed RNA was purified using the zymo RNA purification kit (ZY-R1013) and eluted in 40ul of nuclease free H20. RNA yield was determined using Qubit RNA BR reagents (Thermos Fisher scientific) (typical yield 500-700 ng/ul).

#### guidedNOMe-seq library preparation

After purification of the NOMe treated genomic DNA we quantify DNA concentration using hs/brDNA qubit kit (Thermo Fisher Scientific). For our guidedNOMe-seq libraries the first steps are performed in technical quadruplicates to increase library complexity. (1) 100–150 ng of genomic DNA is dephosphorylated by additional of 1ul rSAP (NEB M0371L), 1.5ul NEB3.1 buffer in a total volume of 15ul. rSAP treatment is performed at 37 °C for 30 min and reaction is thereafter heat inactivated for 10 min at 65 °C. (2) Sample is subsequently digested and a-tailed by addition of Cas9 loaded with the in vitro transcribed sgRNA pool as follows: 0.5ul Cas9 (20uM/ul, NEB M0386M), 700 ng in vitro transcribed sgRNAs, 2.5ul Neb3.1, 1ul 10 mM dATP, 1ul Klenow Fragment (3´ → 5´ exo–) (NEB, M0212L), 1 ul Taq polymerase (NEB M0267L), H2O up to 25ul. Samples are incubated at 37 °C for 12H—> 5 min at 72 °C—> and stored at 4 °C, (3) The technical quadruplicates are merged at this step. 1ul RNAseA (20 mg/ml) is subsequently added, and sample is incubated at 37 °C for 15 min, 1ul protK (20 mg/ml), 1ul 10% SDS and 3 ul H20 is added and incubate at 55 °C for 1 h. Digested DNA is cleaned up by performing 1 V Ampure purification and eluted in 28ul H2O). (4) xGen Methyl UDI-UMI Adapters (IDT) were ligated to the Cas9 digested DNA using the NEBNext® Ultra™ II DNA Library Prep Kit (NEB E7645L) while omitting the end repair step. The ligation reaction: 26.5 ul Cas9 digested DNA, 3.5ul end repair buffer, 15 ul Ligation master mix, 0.5 ul ligation enhancers, 1.25ul 1.5uM xGen Methyl UDI-UMI Adapters (IDT). Samples were incubated at 20 °C for 30 min. 0.75 volume Ampure bead purification was performed, and DNA was eluted in 22ul H20. (5) Bisulfite conversion was performed with the EZ DNA METHLYATION-DIRECT™ KIT (Zymo) according to manufacturing recommendations. (6) Bisulfite converted DNA is thereafter amplified using KAPA HiFi Uracil + (Roche) in combination with P5 and P7 specific primers (see Table 1 for oligo sequences). We were having issues with the presence of adapter dimers in our PCR reaction. To overcome this, we performed 12 PCR cycles followed by a 0.6 V Ampure clean-up to remove adapter dimers. Subsequently, the sample was subjected to an additional 6–9 cycles of PCR again followed by a 0.6 V Ampure clean-up. Size distribution and DNA concentration was checked by running a DNA High Sensitivitybioanalyzer Chip (Agilent) and by dsDNA HS Qubit measurements (Thermo Fisher Scientific). Final libraries were pooled and sequenced on an Illumina MISeq in 300 bp PE mode.

#### Amplicon NOMe

Primers pairs for selected ROI were manually designed (see Table 1 for oligo sequences). ROI were amplified from bisulfite converted NOMe treated DNA using KAPA HiFi Uracil + (Roche). PCR products were purified using the QIAquick PCR Purification Kit (Qiagen) and equimolarly pooled. Sequencing libraries were generated using the NEBNext® Ultra™ II DNA Library Prep Kit and sequenced on an Illumina MISeq.

#### Cell culture

Mouse embryonic stem cells (129 × C57BL/6 background) were cultured on gelatin-coated dishes in ES medium containing DMEM (GIBCO 21969–035), supplemented with 15% fetal bovine serum (FBS; GIBCO), 1 × non-essential amino acids (GIBCO), 1 mM sodiumpyruvate (GIBCO), 2 mM l-glutamine (GIBCO), 0.1 mM 2-mercaptoethanol (Sigma), 50 mg/ml penicillin, 80 mg/ml streptomycin, 3 mM glycogen synthase kinase (GSK) inhibitor (Calbiochem, D00163483), 10 mM MEK inhibitor (Tocris, PD0325901), and homemade LIF, at 37 °C in 5% CO2.

#### Genome editing

For homozygous endogenous tagging, an Avi-3xFLAG-FKBP12(F36V)-mNeonGreen tag was introduced at the Adnp locus and a FKBP12(F36V)-2xHA tag was inserted at the Ctcf gene, both c-terminally. The Adnp^lys407^ mutant was generated by inserting a homology donor at the endogenous Adnp locus leading to a frameshift after Valine 406, insertion of a 3xFLAG-V5 tag and premature termination of the Adnp ORF. This mutation behaves like an Adnp knock-out and mimics a human patient mutation Adnp-Lys408Valfs*31 leading to Helsmoortel-van-der-Aa syndrome [49].

The gBlocks encoding the homology donor constructs for the above genome editing were ordered from IDT, dissolved in H2O and cloned in a modified version of the pCRIS-PITCh plasmid [50] using NEBuilder (cat# NEB E2621S).

Adnp was homozygously tagged in mESCs by transfecting a mixture containing 400 ng each of the TALEN plasmids [32], the targeting vector and a plasmid expressing mCherry using Lipofectamine 3000 (thermoFisher Scientific). Ctcf^FKBP12(F36V)−2xHA^ and Adnp^lys407^were generated by transfecting a mixture containing 400 ng Cas9-p2A-mCherry and 800 ng of the targeting vector using Lipofectamine 3000 (thermoFisher Scientific). 2–3 days after transfection 96 single cells were FACS sorted (mCherry high) into a 96-well plate. Single clones were genotyped by PCR, and correct homozygous insertion was confirmed by sanger sequencing and western blot analysis. gRNA oligos, homology donor constructs and cell lines used can be found in Tables 1 and 2.

**Table 2:** Cell lines used in this study

**Name**	**Genotype**	**Clone**
wt_1	mES159-*Rosa26*^*(tg)Cre-ERT2/(tg)BirA-V5*^	
wt_2	mES159-*Rosa26*^*(tg)Cre-ERT2/(tg)BirA-V5*^ *Cbx3*^*FLAG-Avi/FLAG-Avi*^	clone 32
Adnp-3FV5 1	mES159-*Adnp*^*3xFLAG-V5/3xFLAG-V5*^	clone 1.2
Adnp-3FV5 2	mES159-*Adnp*^*3xFLAG-V5/3xFLAG-V5*^	clone 6F
Adnp KO 1	mES159-*Adnp*^Lys408Valfs*31-3xFLAG-V5/Lys408Valfs*31-3xFLAG-V5^	clone 2B
Adnp KO 2	mES159-*Adnp*^Lys408Valfs*31-3xFLAG-V5/Lys408Valfs*31-3xFLAG-V5^	clone 6F
CTCF-dTAG 1	mES159-*Ctcf*^*FKBP12F36V-2HA/FKBP12F36V-2HA*^ Adnp^*3FLAG-V5/3FLAG-V5*^	clone 8a
CTCF-dTAG 2	mES159-*Ctcf*^*FKBP12F36V-2HA/FKBP12F36V-2HA*^ Adnp^*3FLAG-V5/3FLAG-V5*^	clone 9c
Adnp-dTAG	mES159-*Adnp*^*Avi-3xFLAG-FKBP12(F36V)-mNeonGreen/Avi-3xFLAG-FKBP12(F36V)-mNeonGreen*^	clone 1.1
**Name**	**Description**	
wt_1	ES cells with heterozygous knock-in of BirA and Cre-ERT into Rosa26 locus	
wt_2	ES cells with heterozygous knock-in of BirA and Cre-ERT into Rosa26 locus and homozygous c-terminal FLAG-Avi tag on endogenous Cbx3.	
Adnp-3FV5 1	ES cells with endogenous c-terminal 3xFLAG-V5 tag on Adnp (homozygous)	
Adnp-3FV5 2	ES cells with endogenous c-terminal 3xFLAG-V5 tag on Adnp (homozygous)	
Adnp KO 1	ES cells with homozygous truncation of Adnp at lysine 407 leading to premature termination and an Adnp fragment that does not bind chromatin similar to a full knock-out.	
Adnp KO 2	ES cells with homozygous truncation of Adnp at lysine 407 leading to premature termination and an Adnp fragment that does not bind chromatin similar to a full knock-out.	
CTCF-dTAG 1	ES cells with endogenous c-terminal 3xFLAG-V5 tag on Adnp and a c-terminal FKBP12(F36V)-2xHA tag on Ctcf, both homozygous.	
CTCF-dTAG 2	ES cells with endogenous c-terminal 3xFLAG-V5 tag on Adnp and a c-terminal FKBP12(F36V)-2xHA tag on Ctcf, both homozygous.	
Adnp-dTAG	ES cells with a homozygous c-terminal Avi-3xFLAG-FKBP12(F36V)-mNeonGreen tag on endogenous Adnp.	
**Name**	**Origin**	**Internal reference**
wt_1	Ostapcuk et al, 2018	cMB063
wt_2	Ostapcuk et al, 2018	cMB254
Adnp-3FV5 1	Kaaij et al, 2019	
Adnp-3FV5 2	Kaaij et al, 2019	
Adnp KO 1	this study	
Adnp KO 2	this study	
CTCF-dTAG 1	this study	
CTCF-dTAG 2	this study	
Adnp-dTAG	this study	

#### guidedNOMe-seq timecourse

ADNP depletion and recovery time course combined with flow cytometry (BD LSRII SORP flow cytometer) and NOMe profiling was done as follows: 2–4 × 10^5^ Adnp-avi-3x-FLAG-FKBP12^F36V^ mNeon-Green mESC were seeded per 6-well plate well. dTAG-13 compound was added at final concentration of 0.5 μM (Tocris, 1:1000 dilution of 0.5 mM stock solution in DMSO). Fluorescence intensity, as a proxy for ADNP abundance, was measured at the indicated time points in biological duplicates. For the wash-out time course, mESC were cultured for 24 h in the presence of 0.5uM dTAG-13 compound. After 24 h cells were washed with PBS, dissociated from the culturing dish by incubation with TrypLE™ Express (Thermo fisher Cat# 12,605,010) and washed 3 times using culturing medium. Cells were thereafter seeded at appropriate density and processed at the indicated time points. For accurate FACS analysis of Adnp levels, cells were briefly fixed in 4% Paraformaldehyde for 5 min at room temperature and then stored in PBS at 4ºC. Timepoints indicated throughout the Adnp depletion timecourse indicate the moment at which we started harvesting the cells. The actual NOMe profiling of the nuclei started ~ 15 min after the start of the harvesting.

### Computation

#### Design ROI plus sgRNAs

We used the CTCF ChIP-seq peaks and Ctcf motif files from our previous work [33]. REST ChIP-seq data generated by Baricic et al. were downloaded from GEO (GSE112136) [51] and peaks were called using MACS [52]. We used bedtools to count the number of motifs under a peak and retained only peaks with a single motif for the guidedNOMe-seq [53].

sgRNA design was performed using CRISPRseek [54]. The TF binding motif was taken as center of the ROI and extended up and downstream by 300 bp. sgRNA were identified in the first and last 80 bp separately. Only sgRNAs with a predicted off-target score < 25 were retained for further consideration. ROI for which at least 1 sgRNA in the up and downstream 80 bp could be designed were retained. When there were > 1 sgRNAs targeting a single 80 bp region two sgRNAs with the highest predicted efficacy were kept. When only one sgRNA could be designed in the 80 bp region it was retained twice in the final oligo pool. In case a sgRNA started with a Guanine we removed it, because we add two Guanines in the T7 promoter and NEB recommends two Guanines as three may result in 5’ transcript heterogeneity. Next the final oligo was assembled by addition of the T7 promoter (including two guanines) and the sgRNA scaffold. aagcTAATACGACTCACTATAGG––-sgRNA sequence––-GTTTTAGAGCTAGAAATAGCAAGTTAAAATAAGGCTAGTCCGTTATCAACTTGAAAAAGTGGCACCGAGTCGGTGCTTTT.

#### guidedNOMe-seq enrichment

To estimate the enrichement for ROI by guidedNOMe-seq, we first calculated the expected read coverage of all ROI at the given sequencing depth and then divided this by the actual observed read overlap, which revealed a 139 enrichement for replicate 1 and a 100-fold enrichment for replicate 2.

#### guidedNOMe-seq FDR

Here we assumed that REST binding is independent of ChAHP/ADNP activity. To determine the empirical FDR of guidedNOMe-seq we calculated the number of times a REST bound ROI was statistically tested in all our Adnp associated comparisons (Adnp KO vs WT and in our Adnp depletion timecourse) (*n* = 1541) and in how many cases this resulted in a significant change in REST binding (*n* = 11).

#### guidedNOMe-seq suitable ROI

In Fig. 3A we classified ROI suitable for NOMe profiling when all three chromatin quantification windows contained at least 1 GpC, but we advise, if there is the option, to use ROI with as many GpCs ideally spread throughout the ROI.

To determine the number of CTCF bound ROI that are suitable for guidedNOMe-seq we combined two parameters. (1) the presence of GpCs in the three quantification windows (approximately 50%) (Fig. 3A) and (2) the allowed target space to design sgRNAs (ranging from ~ 25% to ~ 74%) (Fig. 3B).

So the lower bound number of suitabel CTCF ROI using these cut-offs is 70,000*0.5*0.25 = 8750 and the upper bound number is 70,000*0.5*0.74 = 25,900.

#### Simple repeat analysis

The annotation of repeat overlap of ROI is based on the Repeat masker table (update 2012–02-07). Overlap was determined using bedtools using the complete ROI length.

#### On- and Off-target score

Off-targets of individual sgRNAs were identified with the CRISPRseek package without modifications [54]. To identify sgRNA on-target scores we used the crisprScore package with default setting [30].

#### Read UMI deduplication and alignment

We used umi_tools (version 1.0.1) to remove reads that contain identical UMIs [55]. To this end we first added the UMI sequences to the read names (using umi_tools extract with the option –bc-pattern = CCCCCCCCNNNNNNNNN). After mapping the reads, we used umi_tools dedup (discarding any unpaired reads) to remove reads that map to the same genomic location and contain identical UMIs. Bisulfite aware read mapping was performed using Biscuit (version 0.3.16) (https://huishenlab.github.io/biscuit/), and the mouse mm10 genome as a reference (with the options -b 1 -f 0 -K NAGATCGGAAGAGCACACGTCTGAACTCCAGTCA -J NAGATCGGAAGAGCGTCGTGTAGGGAAAGAGTGT) and read2 as the first and read 1 as the second input.

For aligning array capture data we used Biscuit (version 0.3.16) [14], and the mouse mm10 genome as a reference (with the options -b 1 -f 0 -J NAGATCGGAAGAGCACACGTCTGAACTCCAGTCA -K NAGATCGGAAGAGCGTCGTGTAGGGAAAGAGTGT) and read 1 as the first and read 2 as the second input. For aligning PCR data, we used Biscuit (version 0.3.16), and the mouse mm10 genome as a reference (with the options -b 0 -f 1 -J NAGATCGGAAGAGCACACGTCTGAACTCCAGTCA -K NAGATCGGAAGAGCGTCGTGTAGGGAAAGAGTGT) and read 2 as the first and read 1 as the second input.

To randomly subsample reads from the alignments (bam files), we used samtools view (version 1.10) [56].

#### GCH methylation calling

To extract the methylation calls in GCH context from the alignment files, we used the get_data_matrix_from_bams function from the fetchNOMe R package (E. Ozonov et al., unpublished, 10.5281/zenodo.8402785). Briefly, this function filters the reads based on mapping quality (we keep reads with mapping quality above 30), bisulfite conversion rate (fragments where the fraction of non GCH and non WCH Cytosines that are methylated is > 0.1 are removed, if there were at least 10 non GCH and non WCG Cytosines present in the fragment). Then it returns a matrix of protection from methylation calls (1 for protected, 0 for not protected) for each GCH position across the whole ROI and each fragment, for each ROI-sample combination. For array capture or PCR data, which do not contain UMIs, we kept only unique (in their genomic positions and methylation pattern across all Cytosines) fragments.

#### NOMe-seq data analysis

All data analysis was performed using R/Bioconductor and a custom made R package (dinoR) and scripts [57].

We first converted the list of GCH protection matrices as returned by the fetchNOMe package into a new NOMe-seq specific data structure: a single cell experiment object that contains information about the ROI in rowData, information about the samples in colData, and several assays. Some assays describe the number of fragments that were analyzed for each ROI, as well as the number of fragments that were filtered out for various reasons (see above). The “reads” assay contains a Genomic Position object with an entry for each position within the ROI, and metadata columns describing the methylation and protection from methylation status for each fragment as a sparse logical matrix. We used *dinoR::metaPlots()* to generate plots of the average protection across ROI of the same type. This function first calculates the mean protection for each position across reads in all ROI-sample combinations, then calculates the mean protection for each sample across ROI of the same type.

To count the number of fragments that represent a certain pattern, *dinoR::footprintCalc()* and *dinoR::footprintQuant()* select 3 windows (default is -50:-25, -8:8, 25:50) around the center of each ROI and calculate the average GpC methylation protection for a given fragment across all GpCs in each window. If it is above 0.5 the window is deemed protected, below 0.5, unprotected. Depending on the protection pattern in all windows, a read is put into one of 5 footprint categories: tf bound (0—1—0), open chromatin (0—0—0), upstream nucleosome (1—1—0), other nucleosome (1—1—1, 1—0—0, 0—0—1, 1—0—1), and downstream nucleosome (0—1—1). For some analyses the three nucleosome categories were combined. If a fragment does not have methylation protection data in all three windows needed for classification, the fragment will not be assigned a pattern.

To find significant differences in pattern abundances between conditions, we used *dinoR::diNOMeTest()*. This function uses *edgeR*::*calcNormFactors* (with TMM normalization) on the total fragment counts per sample to calculate library sizes, which are then used as library sizes for each sample-pattern combination [58]. After estimating dispersions we used *edgeR::glmQLFit* to fit a quasi-likelihood negative binomial generalized log-linear model to the pattern counts, and conducted statistical tests for each ROI, checking for differences in abundance between wild type and knock-out or dTAG treated samples for each pattern fragment count compared to the total fragment counts. *p*-value correction was performed using the Benjamini–Hochberg method.

In addition, we used *dinoR::footprintPerc()* and *dinoR::fpPercHeatmap()* to calculate the percentage of fragments in each pattern, cluster the ROI based on pattern percentages (using Euclidian distance and the ward.D2 method), and draw a heatmap for all samples of percentages across patterns and ROI.

#### Price comparison guided and oligo capture

To compare the price between custom guidedNOMe vs custom oligo capture we took a simplistic approach. In both cases we toke the catalogue price of the lowest synthesis scale and only compared the price from one vendor (Twist bioscience from December 2022). For the guidedNOMe we assumed 4 sgRNA encoding oligo’s of 123 bp length per ROI. For the oligo capture we assumed 1 × tiling and in case of TWIST capture oligo’s this means we need 5 oligo’s of 120 bp to target 600 bp of genomic DNA.

#### ChIP-seq data analysis

To split the ADNP bound ROI into three groups based on Ctcf binding, we used *Rsubread::featureCounts()* to determine the number of CTCF ChIP reads overlapping the innermost 300 bp of the ROI, and calculated counts per million based on the total number of mapped reads [59]. We used only uniquely mapping reads and counted only the 5’ ends of each read after shifting downstream by 80 bp. We then split all ROI with a CTCF motif into three groups based on quantiles of enrichment.

### Supplementary Information

Supplementary Material 1.

Supplementary Material 2: Figure S1. Benchmarking guidedNOMe-seq. (A) Scatter plot comparing fragment number over ROI between replicates of guided NOMe-seq libraries (B) Scatter plot comparing fragment number over ROI before and after UMI correction (C) Scatter plot comparing fragment number over ROI between replicates of two array capture NOMe-seq libraries (D) The percent of mapped reads that cover the assay specific target regions between 3 different target enrichment approaches. Array capture data from Sönmezer et al (E)Density plot showing the percentage of informative (spanning the three chromatin state quantification windows) reads when performing Array capture and guidedNOMe-seq. (F) Line graph comparing oligo synthesis prices when performing either array capture NOMe or guidedNOMe targeting different numbers of loci, as indicated. Figure S2. Basic NOMe-seq analysis. (A) Scatter plot showing the sgRNA on target scores predicted by 6 different algorithms, as indicated versus the observed associated ROI read counts Violin plots showing the sgRNA on target scores predicted by. (B) Line graphs showing the running mean smoothed levels of simple repeats and low complexity regions in the 1500 ROI ordered by the observed fragment counts per ROI. (C) Genome browser view of a ROI showing from top to bottom: (top) the position of the 4 sgRNAs designed up and downstream of the ROI, (middle) guidedNOMe-seq read coverage of the intended ROI flanked by the sgRNAs (left) and background reads originating most likely from Cas9 cutting at the 5’ end and DNA breakage at the 3’ end (right) (D) Barplot showing presence of off target reads that can be linked to predicted off target sgRNA target sites. Individual bars show read linkage split on sgRNA off targets with and without PAM sequence and random controls, as indicated. (E) Shows correlation plots of two wild type replicates depicting the average observed GpC protection per position between the 4x10^6 reads and all other subsampled amounts, as indicated. ROI TF relationship is highlighted by different colors, as indicated (F) Scatterplots showing the reproducibility between replicates of the TF chromatin state classification. Every ROI is depicted as a single dot. We subsampled the reads, as indicated above the average plot. ROI with two or more informative fragments are shown. Figure S3. Quantification of chromatin state changes using guidedNOMe-seq upon ChAHP perturbation. (A) guidedNOMe-seq example of a CTCF binding site and its surrounding (~250bp +/-). Top plot shows the average protection value of the GpCs at the indicated location. Bottom plot shows protected and unprotected state of the GpCs in individual DNA molecules. DNA molecules are sorted based on the chromatin state they are in, as indicated in the barplot on the right. Single ROI plots were generated under wild type conditions and after 24H Ctcf depletion, as indicated. Putative nucleosome protected regions, here assumed to results in stretches of DNA larger then >100bp protected from methylation by M.CviPI have been manually highlighted in grey (B) guidedNOMe-seq example of a CTCF-Adnp co-bound ROI and its surrounding (~250bp +/-). Top plot shows the average protection value of the GpCs at the indicated location. Bottom plot shows protected and unprotected state of the GpCs in individual DNA molecules. DNA molecules are sorted based on the chromatin state they are in, as indicated in the barplot on the right. Single ROI plots were generated under wild type and Adnp-/- conditions, as indicated. (C) Scatterplot showing changes in chromatin state between wild type and Adnp-/- conditions. ROI are colored on TF (REST, CTCF no ADNP and CTCF with ADNP), see right side of the plots. Every symbol is a ROI and the x-axis and y-axis show the fraction of DNA molecules under wild type and Adnp-/- conditions that is in the chromatin state indicated on top of the plot. edgeR was used to identify ROI that significantly change between wild type and Adnp-/- conditions and the shape of the individual ROI corresponds to the edgeR output as indicated on the right hand site of the plots. (D) Scatterplot showing changes in chromatin state between wild type and Ctcf depleted conditions. ROI are colored on TF (REST, CTCF no ADNP and CTCF with ADNP), see right side of the plots. Every symbol is a ROI and the x-axis and y-axis show the fraction of DNA molecules under wild type and Ctcf depleted conditions that is in the chromatin state indicated on top of the plot. edgeR was used to identify ROI that significantly change between wild type and Ctcf depleted conditions and the shape of the individual ROI corresponds to the edgeR output as indicated on the right hand site of the plots.  (E) Stacked barplot showing the three different ROI groups and the amount and direction of significantly changed chromatin states as determined by edgeR between the wild type and Adnp-/-.

## Data Availability

Datasets availability ChIP-seq datasets have been previously published and are accessible at GEO: GSE125129 and GSE97945. The datasets supporting the conclusions of this article are available in the GEO repository: GSE249662 (ChIP-seq) and GSE249661 (NOMe-seq).
