# Supplementary figures and images for: guidedNOMe-seq quantifies chromatin states at single allele resolution for hundreds of custom regions in parallel
Source: BMC Genomics. 2024 Jul 29;25:732. doi: 10.1186/s12864-024-10625-3 (PMC11288131; doi:10.1186/s12864-024-10625-3)

Figure S1

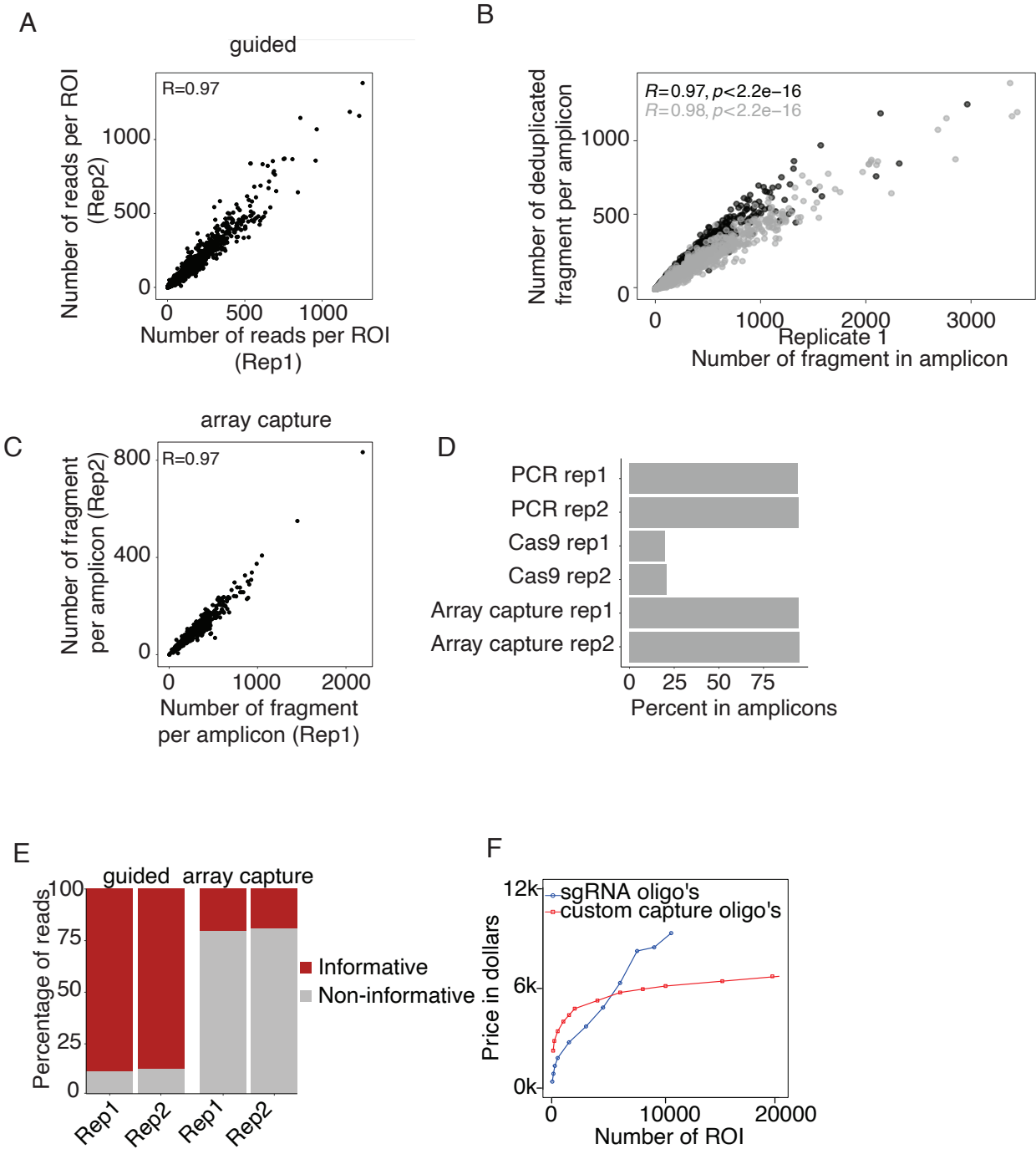

Figure S2

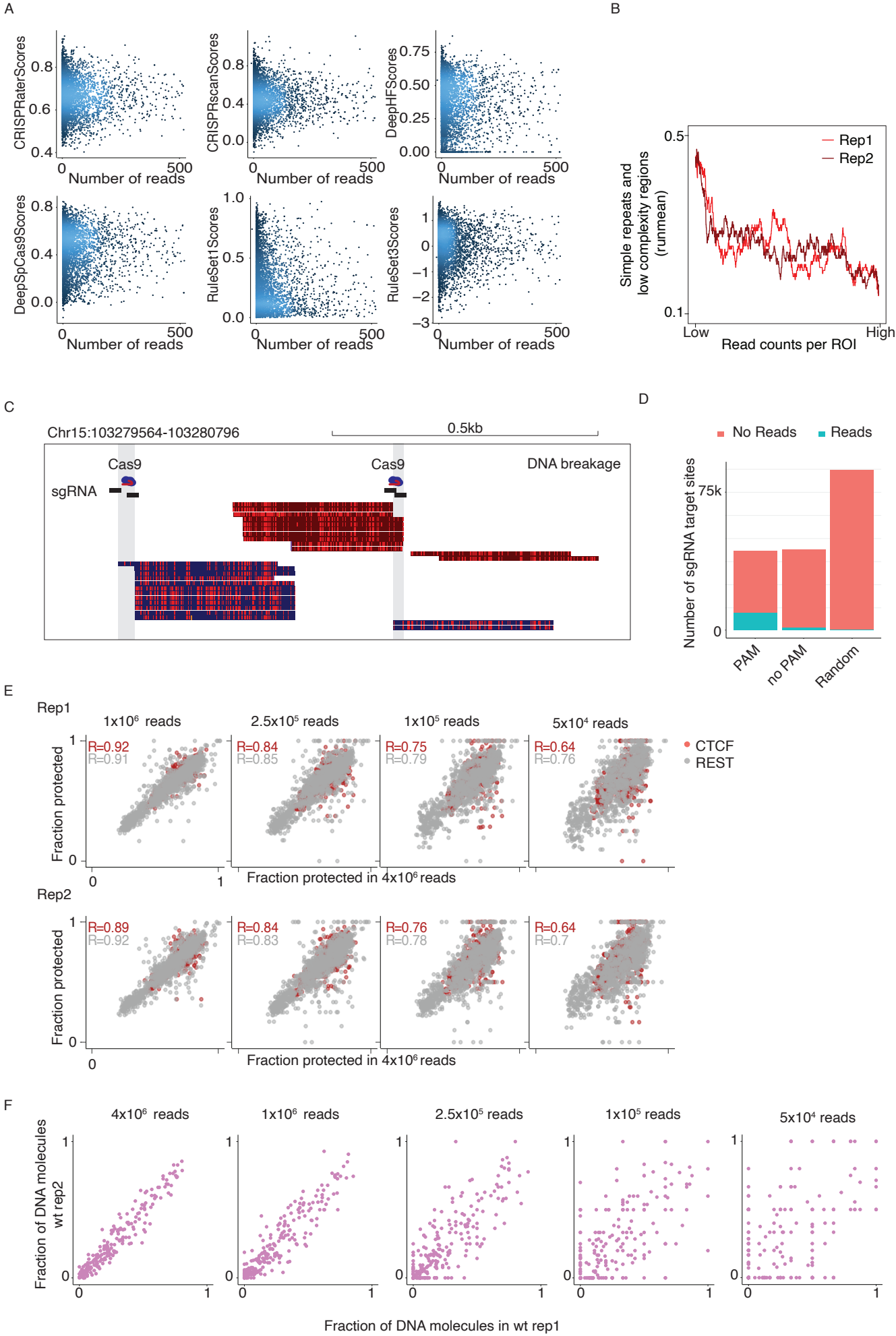

**Figure S3**

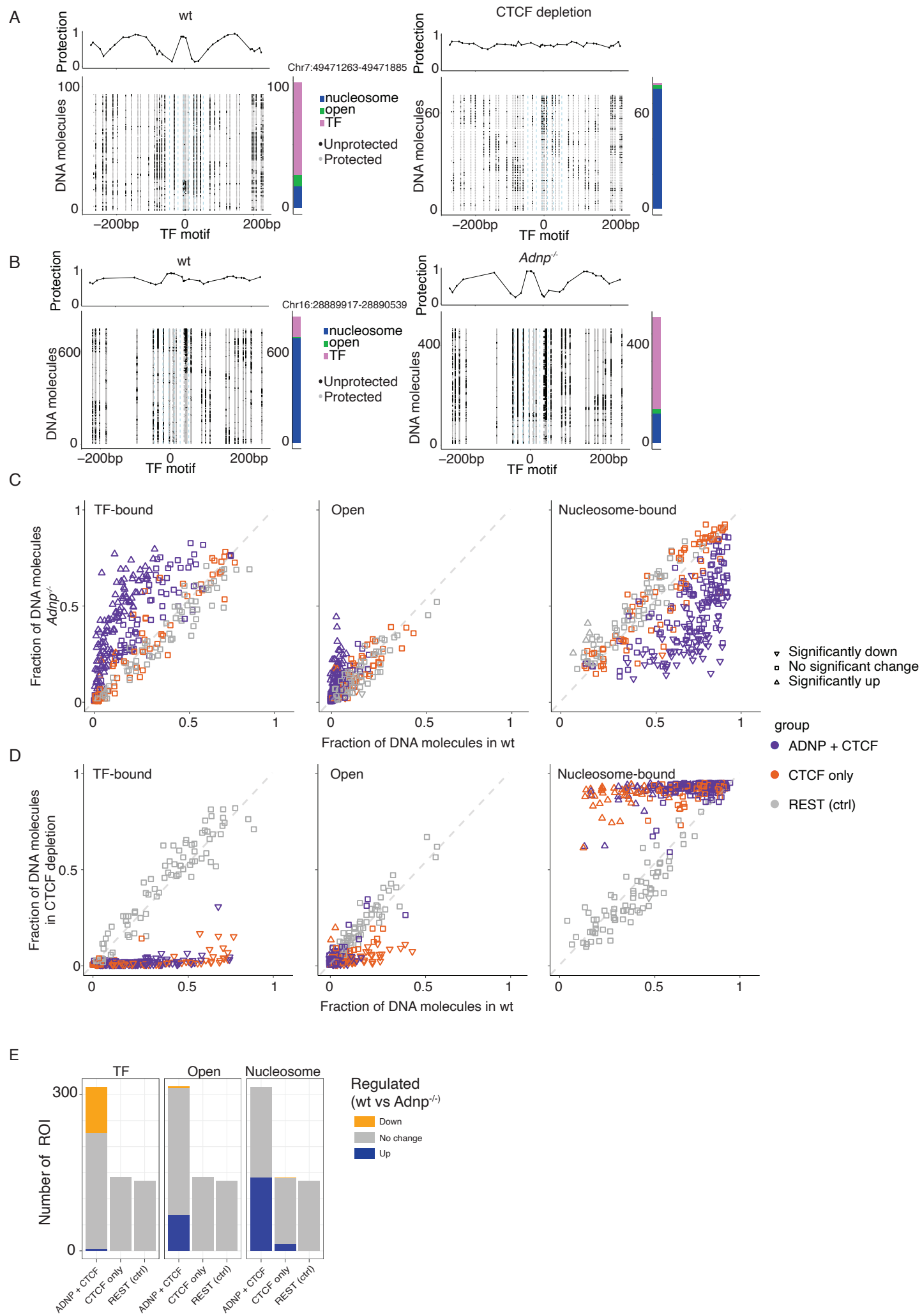

**Figure S4**

**A**

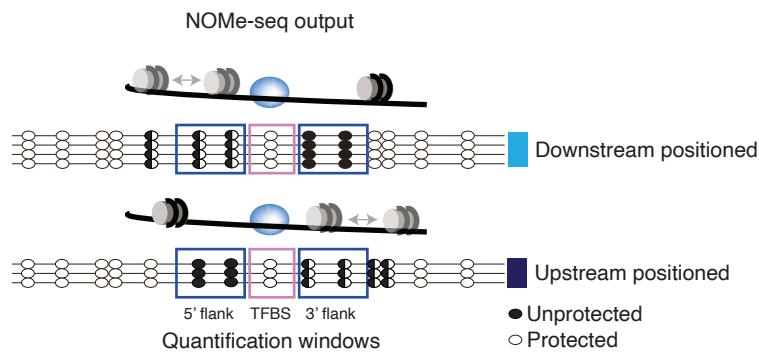

**C**

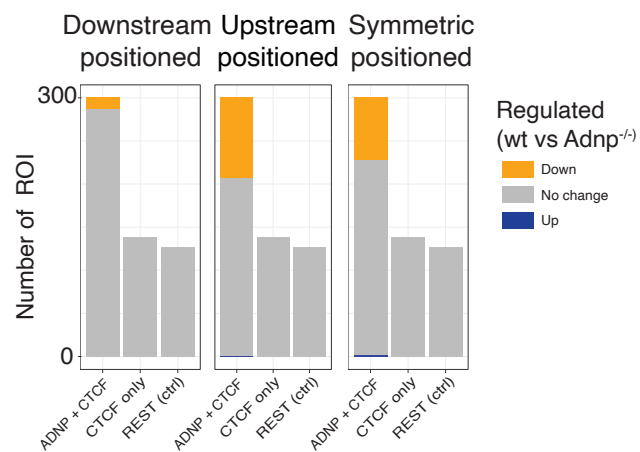

**B**

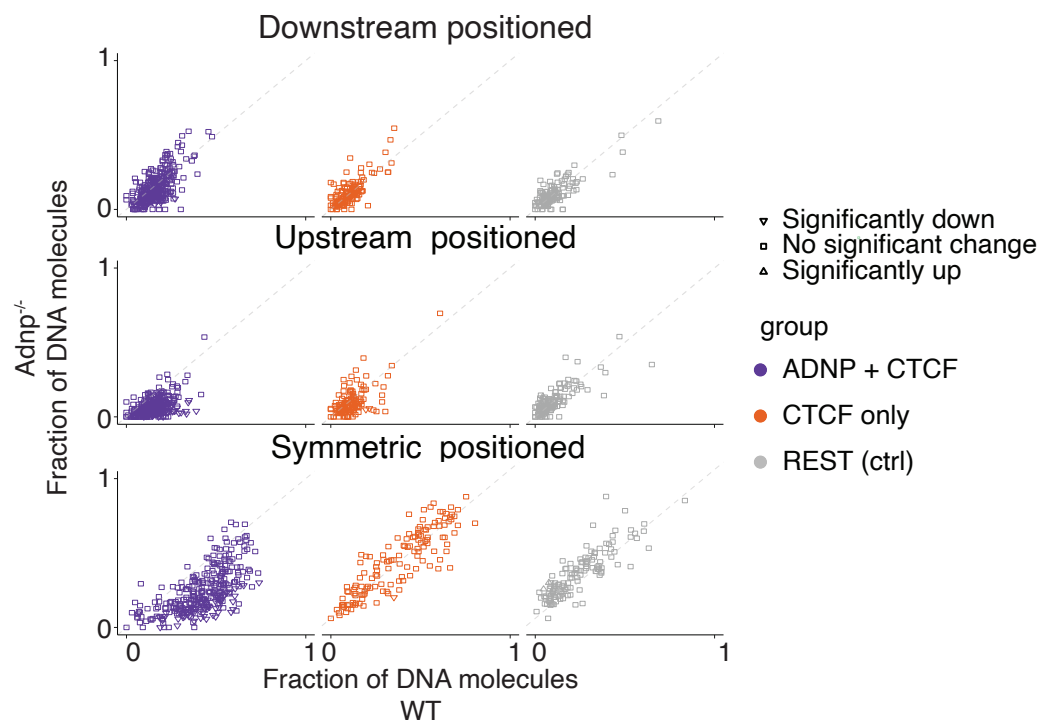

**D**

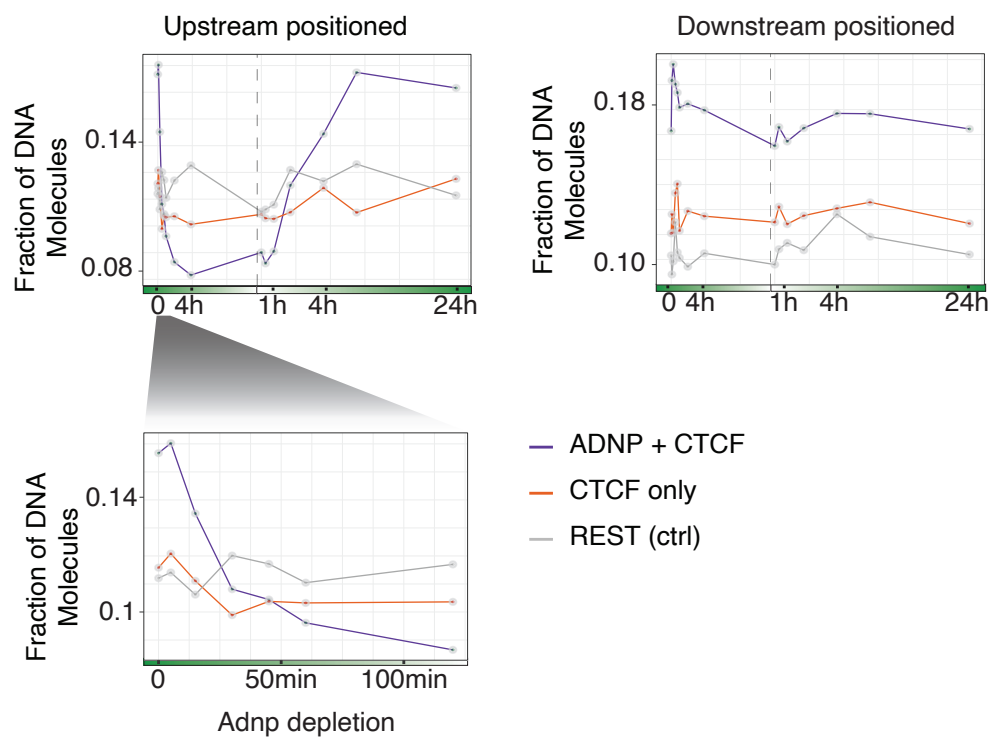

Supplement: Supplementary file 2 — Supplementary Material 2: Figure S1. Benchmarking guidedNOMe-seq. (A) Scatter plot comparing fragment number over ROI between replicates of guided NOMe-seq libraries (B) Scatter plot comparing fragment number over ROI before and after UMI correction (C) Scatter plot comparing fragment number over ROI between replicates of two array capture NOMe-seq libraries (D) The percent of mapped reads that cover the assay specific target regions between 3 different target enrichment approaches. Array capture data from Sönmezer et al (E)Density plot showing the percentage of informative (spanning the three chromatin state quantification windows) reads when performing Array capture and guidedNOMe-seq. (F) Line graph comparing oligo synthesis prices when performing either array capture NOMe or guidedNOMe targeting different numbers of loci, as indicated. Figure S2. Basic NOMe-seq analysis. (A) Scatter plot showing the sgRNA on target scores predicted by 6 different algorithms, as indicated versus the observed associated ROI read counts Violin plots showing the sgRNA on target scores predicted by. (B) Line graphs showing the running mean smoothed levels of simple repeats and low complexity regions in the 1500 ROI ordered by the observed fragment counts per ROI. (C) Genome browser view of a ROI showing from top to bottom: (top) the position of the 4 sgRNAs designed up and downstream of the ROI, (middle) guidedNOMe-seq read coverage of the intended ROI flanked by the sgRNAs (left) and background reads originating most likely from Cas9 cutting at the 5’ end and DNA breakage at the 3’ end (right) (D) Barplot showing presence of off target reads that can be linked to predicted off target sgRNA target sites. Individual bars show read linkage split on sgRNA off targets with and without PAM sequence and random controls, as indicated. (E) Shows correlation plots of two wild type replicates depicting the average observed GpC protection per position between the 4x10^6 reads and all other subs [file 12864_2024_10625_MOESM2_ESM.pdf]
